# Mapping spatially resolved transcriptomes in human and mouse pulmonary fibrosis

**DOI:** 10.1038/s41588-024-01819-2

**Published:** 2024-07-01

**Authors:** Lovisa Franzén, Martina Olsson Lindvall, Michael Hühn, Victoria Ptasinski, Laura Setyo, Benjamin P. Keith, Astrid Collin, Steven Oag, Thomas Volckaert, Annika Borde, Joakim Lundeberg, Julia Lindgren, Graham Belfield, Sonya Jackson, Anna Ollerstam, Marianna Stamou, Patrik L. Ståhl, Jorrit J. Hornberg

**Affiliations:** 1 Safety Sciences, Clinical Pharmacology and Safety Sciences, R&D, AstraZeneca, Gothenburg, Sweden; 2 Department of Gene Technology, KTH Royal Institute of Technology, Science for Life Laboratory, Stockholm, Sweden; 3 Translational Science and Experimental Medicine, Research and Early Development, Respiratory and Immunology, BioPharmaceuticals R&D, AstraZeneca, Gothenburg, Sweden; 4 Pathology, Clinical Pharmacology and Safety Sciences, R&D, AstraZeneca, Cambridge, UK; 5 Quantitative Biology, Discovery Sciences, R&D, AstraZeneca, Gothenburg, Sweden; 6 Animal Science and Technology, Clinical Pharmacology and Safety Sciences, R&D, AstraZeneca, Gothenburg, Sweden; 7 Bioscience In Vivo, Research and Early Development, Respiratory and Immunology, BioPharmaceuticals R&D, AstraZeneca, Gothenburg, Sweden; 8 Translational Genomics, Centre for Genomics Research, Discovery Sciences, R&D, AstraZeneca, Gothenburg, Sweden; 9 Late-Stage Development, Respiratory and Immunology, BioPharmaceuticals R&D, AstraZeneca, Gothenburg, Sweden

**Keywords:** Respiratory tract diseases, Gene expression, Experimental models of disease, Translational research, RNA sequencing

## Abstract

Idiopathic pulmonary fibrosis (IPF) is a progressive lung disease with poor prognosis and limited treatment options. Efforts to identify effective treatments are thwarted by limited understanding of IPF pathogenesis and poor translatability of available preclinical models. Here we generated spatially resolved transcriptome maps of human IPF (*n* = 4) and bleomycin-induced mouse pulmonary fibrosis (*n* = 6) to address these limitations. We uncovered distinct fibrotic niches in the IPF lung, characterized by aberrant alveolar epithelial cells in a microenvironment dominated by transforming growth factor beta signaling alongside predicted regulators, such as TP53 and APOE. We also identified a clear divergence between the arrested alveolar regeneration in the IPF fibrotic niches and the active tissue repair in the acutely fibrotic mouse lung. Our study offers in-depth insights into the IPF transcriptional landscape and proposes alveolar regeneration as a promising therapeutic strategy for IPF.

## Abstract (web summary)

Spatially resolved transcriptome analysis of human and mouse idiopathic pulmonary fibrosis identifies disease-associated niches and a role for aberrant alveolar epithelial cells in human disease pathogenesis.

## Main

Idiopathic pulmonary fibrosis (IPF) is a chronic lung disease characterized by progressive and irreversible scarring of the lung. Treatment options are limited, and the development of new therapies is impeded by incomplete understanding of disease pathogenesis and translatability limitations of available preclinical models. Recent advances into mechanistic understanding of IPF pathogenesis reveal complex gene-environment interactions as key pathophysiological drivers^1–3^.

Single-cell studies have revealed IPF-associated cell states, including atypical epithelial cells, fibroblasts^4,5^ and profibrotic alveolar macrophages^6,7^. Interestingly, a novel *KRT5*^−^/*KRT17*^*+*^ aberrant basaloid (AbBa) epithelial cell population has been independently identified in multiple studies^4,5,8,9^, expressing epithelial, basal and mesenchymal markers and genes related to senescence and extracellular matrix (ECM) production. These cells probably originate from alveolar type 2 (AT2) or club cells^4,5,9,10^, but their role in the fibrotic microenvironment remains elusive. A closely related *Krt8*^*+*^ alveolar differentiation intermediate (ADI) cell population is present in the widely used mouse model of bleomycin (BLM)-induced pulmonary fibrosis^11^, which, in contrast to the IPF lung, features relatively rapid inflammatory onset, epithelial regeneration and fibrosis resolution^12^.

Although recent single-cell RNA sequencing (scRNA-seq) studies have substantially advanced our understanding of the IPF lung cellular composition^4–7,13–15^, they lack insights into tissue architecture and cellular interplay in a spatial context. Spatially resolved transcriptomics (SRT) enables RNA profiling of intact tissue^16–19^ and can illuminate dynamic cellular interactions in the lung^20–22^. However, a transcriptome-wide map of extensive areas of the fibrotic lung is currently missing.

We applied SRT to map the fibrotic lung in human IPF and the BLM mouse model. SRT was integrated with scRNA-seq data to characterize the AbBa cell microenvironment and delineate the dynamic crosstalk between alveolar epithelial cells, myofibroblasts, fibroblasts and profibrotic macrophages. These spatial atlases broaden our understanding of the IPF cellular interplay and unveil key convergent and divergent pathways in human IPF and the BLM mouse model.

## Results

### Spatial transcriptomics of healthy and IPF human lungs

**Fig. 1:**
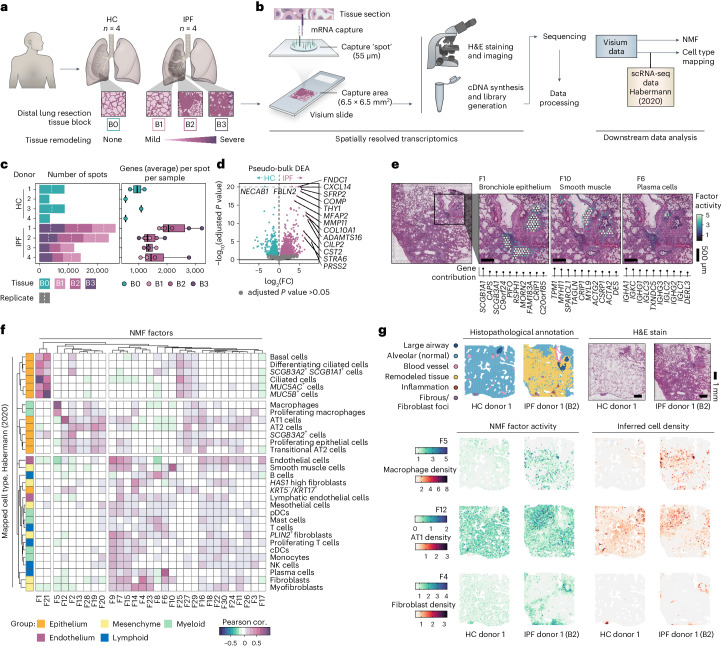
Spatial transcriptomic profiling of human pulmonary fibrosis. **a**, Tissue sections from distal lung resection samples from HCs (B0; *n* = 4) and patients with IPF (*n* = 4), were sectioned and analyzed using the Visium Spatial Gene Expression technology. Three tissue blocks exhibiting progressive tissue remodeling (B1–3) were selected from each IPF donor. **b**, Schematic illustration of the Visium workflow and subsequent data processing steps. NMF was used for dimensionality reduction, generating 30 distinct factors. Cell-type distributions were inferred through integration with a scRNA-seq dataset published by Habermann et al.^5^ (2020; GSE135893). **c**, Summarizing descriptions of the data, including the number of Visium capture spots per sample and the average number of unique genes detected per spot and sample. The box plot center line is the median, the box limits are the upper and lower quartiles, the whiskers are 1.5× interquartile range and the points are the value per Visium sample. **d**, Pseudobulk DEA comparing pooled HC and IPF Visium data per donor to identify significant DEGs between conditions based on data from entire tissue sections. **e**, Spatial distribution maps for selected NMF factors that correspond histological and/or transcriptional profiles of bronchiole epithelium (F1), smooth muscle (F10) and plasma cells (F6). **f**, Pearson correlation (cor.) heat map of NMF factor activity and inferred cell-type densities, using the Habermann et al. scRNA-seq dataset, across all spots in all samples. **g**, Example of histopathological annotations performed on sections from each HC and IPF tissue block based on the H&E-stained Visium sections. Visualizing spatial NMF activity and inferred cell-type densities showcases the colocalization of highly correlated factor-cell pairs. NMF, non-negative matrix factorization; pDC/cDCs, plasmacytoid/classical dendritic cells; NK cells, natural killer cells. Source data

**Extended Data Fig. 1:**
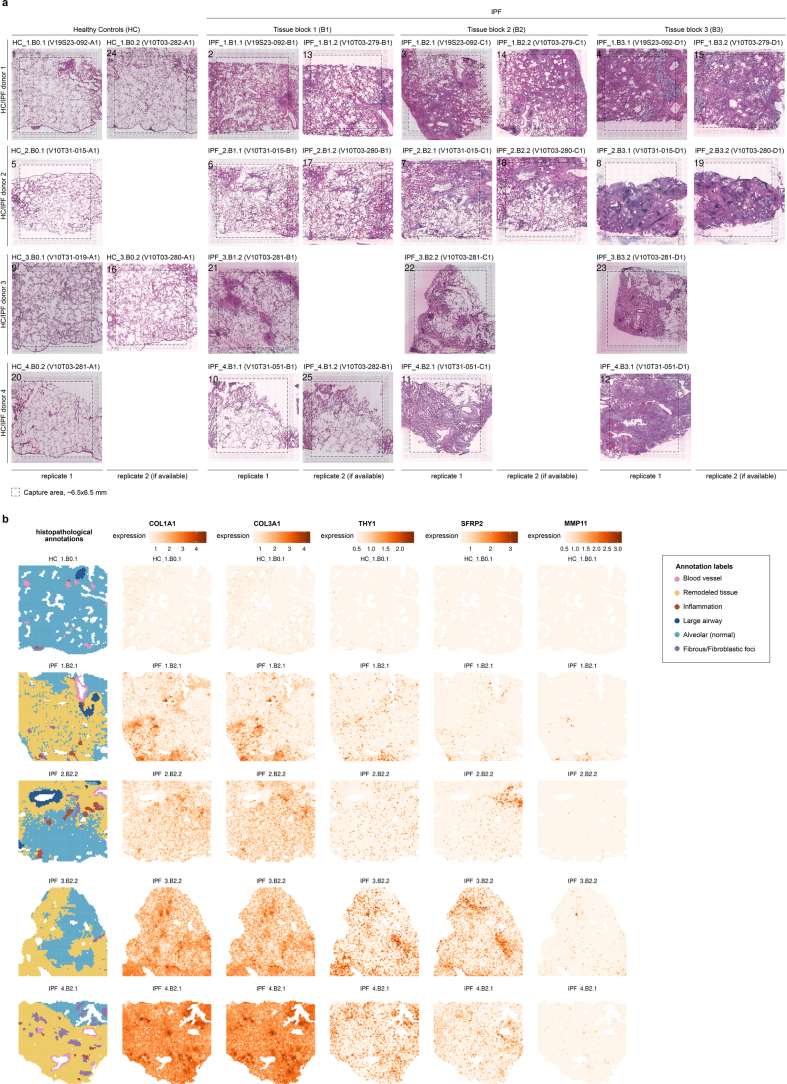
Histological and spatial gene expression analysis of human lung tissues. **a)** Hematoxylin and eosin (H&E) stained sections of lung tissue samples from four healthy controls (HC 1-4; ‘B0’) and four IPF patients (IPF 1-4), used for Visium experiments. Each IPF patient sample includes three samples (‘B1’, ‘B2’, ‘B3’) representing a gradient in the extent of fibrotic injury, from mild to severe tissue remodeling. Visium data from a total of 25 tissue sections were analyzed, including replicate sections for some tissues to control for technical variability. **b)** Histopathological annotations were performed on the H&E-stained sections, here showing the annotations in sections from one HC and each IPF donor, displaying the extent of tissue remodeling. Differential expression analysis performed on pseudo-bulk Visium data between IPF and HC identified several extracellular matrix and fibrosis-related genes, and when plotted spatially, their expression largely originates from regions of tissue fibrosis.

We generated transcriptome-wide spatial profiles of freshly frozen human lung resection samples from four patients with IPF (IPF 1–4, collected during lung transplantation) and four subjects with no known lung disease (healthy controls (HCs) 1–4 and ‘B0’, collected postmortem) using the Visium Spatial Gene Expression platform (Fig. 1a,b and Supplementary Table 1). For each patient with IPF, three tissue blocks (‘B1’, ‘B2’ and ‘B3’) reflecting increasing extent of fibrotic injury within the same donor were selected (Fig. 1a and Extended Data Fig. 1a). The sample selection and study design are further described in Methods and Supplementary Note 1.

**Extended Data Fig. 2:**
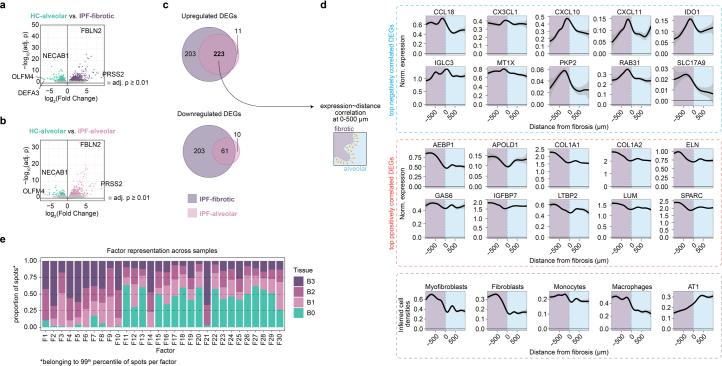
Differential gene expression in IPF and NMF factor distribution. **a)** Differential gene expression between annotated fibrotic regions of IPF lungs and HC samples. **b)** Differential gene expression between annotated alveolar regions from IPF lungs and samples from healthy control (HC) lungs. **c)** Overlap of significantly (FDR-adjusted p-value < 0.01) upregulated (top) and downregulated (bottom) DEGs in IPF alveolar and fibrotic regions compared to HC. **d)** The spatial gene expression of the 223 overlapping upregulated DEGs were used to calculate correlation (Pearson) values from the fibrotic border distances using gene expression against the 0-500 µm distances. The gene expression for the top 10 significantly (Benjamini–Hochberg adj. p < 0.05) up and down correlated genes, as well as the inferred cell densities of selected cell types, were visualized for the 2000 µm distances centered around the fibrotic border using a generalized additive model (GAM) for the regression line. Gray areas correspond to 95% confidence intervals. **e)** NMF factorization was performed on all human Visium samples. Spots with the highest activity (99^th^ percentile; F^hi^) for each factor were identified and used to calculate the proportion of F^hi^ spots among all spots, grouped by tissue block grading (B0-B1) and displayed as a stacked bar chart. NMF, non-negative matrix factorization; DEG, differentially expressed gene; HC, healthy controls. Source data

A total of 255–444 million sequencing reads (average 349 million) per sample were generated (Supplementary Table 1). We analyzed an average of ~4,000 spots per tissue section (each spot representing a transcriptome of the mixture of cells in the tissue that covers the spot), capturing an average of >1,500 unique genes per spot (Fig. 1c). We observed a higher average number of genes per spot and transcript count levels in IPF samples compared with HC, probably due to disease-associated differences in cellular density between conditions. A pseudobulk differential expression analysis (DEA) between IPF and HC samples identified 1,469 differentially expressed genes (DEGs) (Fig. 1d), including genes associated with fibroblasts, previously reported to be upregulated in IPF (*FNDC1*, *COL10A1* and *THY1*)^23^, as well as matrix metalloproteinases^24^ and genes involved in IPF-associated signaling pathways (*SFRP2*, *WNT10A* and *TGFBI*)^25,26^ (Supplementary Table 2). While several upregulated genes in the IPF samples mapped to areas of remodeled fibrotic tissue, alterations were also seen in minimally remodeled alveolar tissue (Extended Data Fig. 1b). These areas were further examined by comparing annotated ‘alveolar’ or ‘fibrotic’ regions within the IPF samples to alveolar regions in the HC samples (Extended Data Fig. 2a,b and Supplementary Note 2). A high number of DEGs were found in fibrotic regions compared with IPF alveolar regions, with 95% of upregulated DEGs in IPF alveolar areas also found in fibrotic regions (Extended Data Fig. 2c and Supplementary Table 3). Among these, many DEGs displayed a relationship between expression and proximity to the fibrotic-alveolar interface (Extended Data Fig. 2d and Supplementary Table 3). At the fibrotic border, multiple chemokine ligands (*CCL18*, *CX3CL1*, *CXCL10* and *CXCL11*) displayed peaked expression that decreased into alveolar regions, suggesting recruitment of immune cells (for example, profibrotic macrophages) to the leading edge of fibrosis. Conversely, ECM-associated genes (*COL1A1*, *COL1A2* and *LUM*) had a decreased expression at the border, while expression was higher in the fibrotic and adjacent alveolar tissue (Supplementary Note 2).

#### Data deconvolution identifies structures and cell types

The data were deconvoluted into 30 ‘factors’ using non-negative matrix factorization (NMF)^27^ (Supplementary Table 4, describing associated genes, pathways and cell types). Each factor was driven by expression of covarying genes with little overlap observed between the top contributing genes for most factors (Supplementary Fig. 1b,c). These factors revealed signatures of distinct cell types and structures including mixed bronchiolar epithelial cell types (factor 1 (F1)), smooth muscle cells (F10) and plasma and B cells (F6) (Fig. 1e). The spatial distribution of cell-type densities was further inferred by integration^28^ with an IPF-derived scRNA-seq dataset^5^ (referred to as ‘Habermann’^5^). This revealed a distinct group (F1 and F21) that correlated with ciliated airway cell types, including basal cells, club cells, ciliated cells and *MUC5B*^*+*^ cells (Fig. 1f), in line with spatial mapping of F1 activity to bronchial epithelium. Other factors correlated specifically with the alveolar compartment (Fig. 1g), including alveolar macrophages (spatial overlap with F5), alveolar type 1 (AT1) cells (spatial overlap with F12 and annotated alveolar tissue) and AT2 cells. An additional group of factors corresponded to immune cells and stromal components, including lymphocytes, endothelial cells and fibroblasts (spatial overlap with F4 and areas labeled as fibrous or remodeled tissue). Several factors could not be clearly attributed to specific cell types or groups, probably representing a more complex mixture of cells, cell types not annotated in the reference dataset, and/or uncharacterized cell states. This included F16 in the alveolar compartment of HC and IPF lungs (Fig. 1f), dominated by prostaglandin signaling genes and AT1, AT2 and fibroblast markers (Supplementary Table 4).

#### Factor activity reveals pathways and cellular interactions

**Fig. 2:**
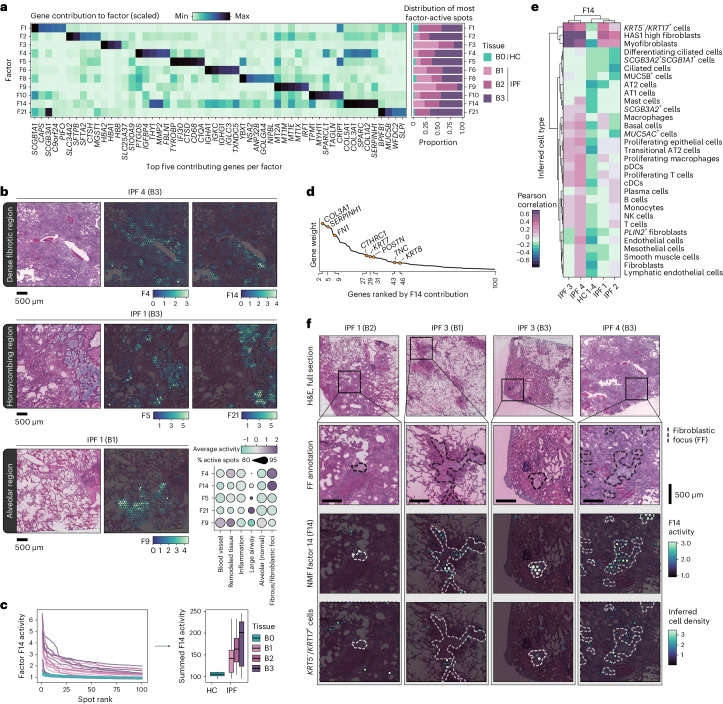
Disease-associated signatures revealed by NMF. **a**, NMF identified signatures over-represented in IPF tissue. Their relative contribution to each factor (scaled) is displayed for the top five contributing genes per factor, and the proportion of spots with the highest activity (99th percentile) by condition and tissue severity grade. **b**, Spatial representation of selected NMF factors across IPF lung sections, demonstrating distinct localization patterns that was observed across all IPF samples. F4 and F14 marked heavily fibrotic regions, F5 and F21 associated with honeycombing structures and F9 were seen in alveolar regions. The dot plot displays the average activity (scaled and centered) and detection rate (percentage of spots with increased activity) within the annotated histological regions across all tissue blocks. **c**, Activity profile of the top 100 ranked spots per sample based on F14 activity (line chart), highlighting a consistent distinction between HC and IPF tissue samples, further summarized by summing the F14 activity levels and grouped based on tissue remodeling extent (B0, *n* = 6; B1, *n* = 7; B2, *n* = 6; B3, *n* = 6) (box plot). The center line is the median, the box limits are the upper and lower quartiles and the whiskers are 1.5× interquartile range. **d**, Ranking of the top 100 genes for F14 based on gene weight (contribution) to the factor, with keratins, collagens and other fibrosis-related genes emphasized. **e**, Correlation heat map between F14 activity and densities of inferred cell types within spatial spots, capturing potential colocalization of F14 and cell types (strong correlation suggests spatial co-occurrence). **f**, Visualization of FF annotations, F14 activity and the distribution of inferred *KRT5*^−^/*KRT17*^+^ cells in selected samples and regions in all IPF donor lungs. Source data

Further examination of factor distribution across samples revealed 11 factors that were more prevalent in IPF compared with HC (Fig. 2a and Extended Data Fig. 2e). These factors are associated with important IPF cell morphologies and processes, including ECM-related pathways, and overlapped regions of fibrosis (F4 and F14) or IPF ‘honeycomb’ formations (F5 and F21) (Fig. 2b). F5 displayed markers of dendritic cells and macrophages, while F21 presented a *MUC5B*-expressing airway epithelial signature (Supplementary Table 4). The F21 profile might reflect a previously identified *MUC5B*^+^, *BPIFB1*^+^ and *SCGB3A1*^+^ IPF-associated cell population^29^. In line with our SRT data, MUC5B expression has previously been localized to honeycomb cysts, and *MUC5B* polymorphisms have been linked to IPF risk^30^. F9 was unique to IPF lungs and characterized by genes related to oxidative stress, inflammation, ECM remodeling and vascular changes. It was moreover particularly present in IPF samples with less dense fibrosis (‘B1’), suggesting involvement in early-stage fibrotic processes (Supplementary Note 3).

Among the ECM/fibrosis-related factors, both F4 and F14 were notably active in IPF tissues, probably reflecting pathological remodeling (Fig. 2c and Supplementary Note 3). In addition to collagens and fibrosis-related genes, the F14 signature also included keratins such as *KRT7* and *KRT8* (Fig. 2d). F14 activity correlated with inferred cell-type densities of *KRT5*^−^/*KRT17*^+^ AbBa cells, myofibroblasts and the recently described *HAS1*-hi fibroblast subtype^5^, specifically in the IPF samples (Fig. 2e). One lung (IPF donor 2) demonstrated a less pronounced correlation between F14 activity and the *KRT5*^−^/*KRT17*^+^ AbBa cell type. This deviation may stem from donor heterogeneity in cellular composition of the studied samples or more general variation in disease manifestations between patients.

Visual inspection confirmed that F14-positive spots coincided with the correlated cell types and revealed that F14 activity spatially aligned with fibroblastic foci (FF) (Fig. 2f), a histological feature of active tissue remodeling^21,31,32^. In many instances, spots with elevated *KRT5*^−^/*KRT17*^+^ AbBa cell densities appeared to be situated along the FF borders, confirming the previously proposed positioning of these cells within the fibrotic human lung^4^. Importantly, our NMF approach thus identified a signature encompassing the *KRT5*^−^/*KRT17*^+^ AbBa cell type independently of scRNA-seq data, placed in its spatial histological context across IPF samples.

### Characterization of the AbBa niche in IPF

**Fig. 3:**
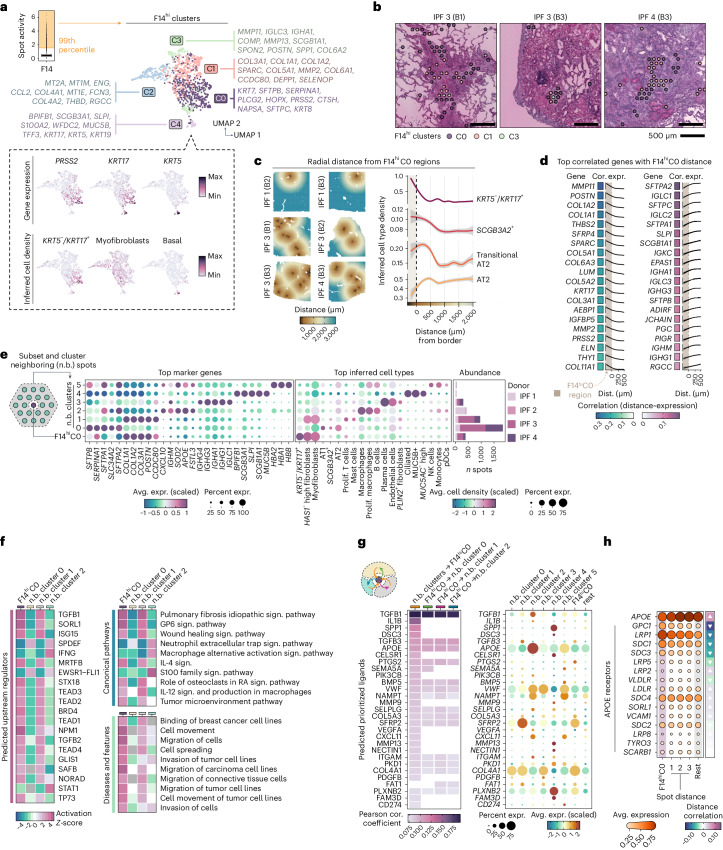
Cellular and molecular deconvolution of the AbBa niche. **a**, Clustering of the 99th percentile of F14 active spots (F14^hi^; yellow shaded area) into five clusters (F14^hi^C0–4), visualized in the UMAP space with the top gene markers listed and expression of AbBa cell markers *PRSS2* and *KRT17* and basal cell marker *KRT5*. The box plot center line is the median, the box limits are the upper and lower quartiles and the whiskers are 1.5× interquartile range. **b**, Spatial location of F14^hi^ clusters within FF in representative IPF tissue sections. **c**, Inferred cell-type densities around F14^hi^C0 boundaries (dashed line), with radial distance. The distance below zero indicatesF14^hi^C0 positive spots (shaded area). **d**, Pearson correlation (cor.) and gene expression (expr.) of top 20 genes (positive and negative) across a 0–500 µm distance (dist.) radius from the F14^hi^C0 border. **e**, Clustering of neighboring F14^hi^C0 spots (~200 µm) into six clusters (n.b. cluster 0–5) with average expression (avg. expr.) of top marker genes and selected average inferred cell-type densities highlighted in the dot plot. The bar chart displays distribution per donor. **f**, Enrichment analysis (IPA) of n.b. clusters 0–2 using marker genes (adjusted *P* < 0.05). The heat maps display activation *Z*-scores of top predicted upstream regulators enriched pathways and diseases and functions. **g**, NicheNet analysis of cell–cell communication showing prioritized ligands acting upon F14^hi^C0 and n.b. clusters 0–2 regions (left) with their average expression (avg. expr.) levels (right). **h**, Average expression of *APOE* and its receptors across F14^hi^C0 and over radial distance (three spots, ~300 µm) and background expression in remaining spots (‘rest’). The directional arrows show correlation (Pearson) trends across spot distance. Prolif. T cells, proliferating T cells; Sign. pathway, signaling pathway. Source data

**Extended Data Fig. 3:**
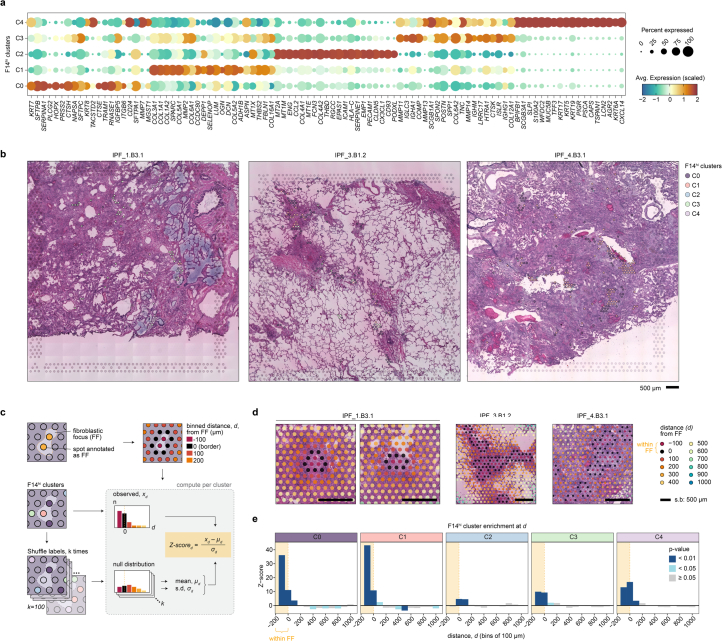
F14^hi^ clusters in human lung tissues. **a)** Gene expression profiles for F14^hi^ cluster (C0-C4) top marker genes. Color scale represents average scaled expression, and dot size reflects the proportion of spots expressing each gene. **b)** Spatial distribution of F14^hi^ clusters C0-C4 overlaid on H&E images of three human IPF lung sections. Similar spatial patterns were observed in all IPF sections. **c)** Approach for calculating spatial enrichment of each F14^hi^ clusters at distances from FF. The radial distances, *d*, from the FF were extracted for all spots, where 0 µm corresponds to the outer FF border. The observed F14^hi^ cluster spot locations were registered at each distance (binned into 100 µm to cover each row of spots), and the F14^hi^ cluster labels were shuffled across all spots within in each sample *k*=100 times to produce a null distribution (*that is*, how each cluster would be distributed if there were no spatial patterns). For each cluster, the observed count at every distance (*x*_*d*_) and the mean (*µ*_*d*_) and standard deviation (s.d., *σ*_*d*_) across all randomization rounds were used to compute a Z-score per *d*. **d)** Spatial overlay of the FF distance-colored spots over the H&E-stained tissue image for a few selected areas. **e)** Enrichment of each F14^hi^ cluster at distances (µm) from FF using a Z-score computed with a randomized spatial cluster distribution as the baseline. Two-sided p-values were calculated using the Z-scores, and the bars are colored based on their significance levels (dark blue: p < 0.01; light blue: p < 0.05). FF, fibroblastic focus; H&E, Hematoxylin and eosin. Source data

To better understand the cell-type heterogeneity in F14, we isolated its most active spots (denoted F14^hi^) and identified five distinct subclusters, F14^hi^ C0–C4 (Fig. 3a and Extended Data Fig. 3a). Defining genes of C0 corresponded to markers of the *KRT5*^−^/*KRT17*^+^ AbBa cell type (for example, *PRSS2* and *KRT7*)^5^, characteristically devoid of the basal cell marker *KRT5*. The remaining four F14^hi^ clusters expressed genes corresponding to fibroblasts and myofibroblasts (C1 and C2), macrophages (C3) and basal and secretory airway epithelial cells (C4). Based on the marker gene profiles, C1 and C2 appeared to represent fibrotic populations with distinct roles. C1 displayed a matrix deposition and scar formation profile whereas C2 had markers indicative of stress responses (metallothioneins), immune modulation (*CCL2* and *FCN3*) and vascular interactions (*ENG* and *THBD*), probably reflecting diverse fibroblast phenotypes within the fibrotic niche of the IPF lungs. F14^hi^ C0 was markedly present in three out of four IPF donors (lowly abundant in donor 2), consistent with AbBa cell detection rates from prior scRNA-seq studies^4,5^ (Supplementary Note 4 and Supplementary Fig. 2). Upon visual inspection, C0 spots were often seen to colocalize with edges of FF (Fig. 3b and Extended Data Fig. 3b). Histopathological annotations of FF-overlapping spots were used to compute the overrepresentation of each F14^hi^ cluster at a distance to the FF borders (Extended Data Fig. 3c,d). This revealed significant enrichment of cluster C0 around the edges and within the FF (Extended Data Fig. 3e). Given the multicellular resolution of Visium, it is challenging to capture the precise lining of FF; however, our results support previous reports of AbBa cell localization at the FF periphery^4,5^. In comparison, the cluster marked by fibroblast genes (C1) was highly concentrated within the FF.

### Crosstalk and signaling in the AbBa microenvironment

We found a higher abundance of AT2 cells and transitional AT2 cells around the F14^hi^C0 AbBa niche, compared with more distant regions (Fig. 3c). Notably, the density of transitional AT2 cells peaked slightly closer to the AbBa niche than AT2 cells, suggesting a possible differentiation lineage from AT2 to transitional AT2 cells and toward AbBa cells, consistent with a previously proposed cell trajectory^5^, captured in space. Additionally, the proximity of *SCGB3A2*^+^ secretory cells to F14^hi^C0 spots aligns with previous findings suggesting them as another potential source for AbBa cells^5,29^.

We observed a decline in matrix remodeling and fibrosis-associated genes (for example, *MMP11*, *POSTN* and *COL1A2)* with increasing distance from F14^hi^C0 (Fig. 3d), indicating elevated fibrotic activity around AbBa cells. Conversely, genes linked to alveolar function and immune response (for example, *SFTPA2*, *SFTPC* and *SLPI)* showed lower expression within C0 compared with its immediate surroundings. A group of immunoglobulin-related genes (for example, *IGLC1*, *IGKC* and *PIGR*) were expressed near AbBa cell-dense areas but not within, implying a differential immune response or possible exclusion of certain immune elements from the AbBa microenvironment.

Analysis of areas neighboring (n.b.) the F14^hi^C0 spots identified subclusters containing alveolar epithelial cells (n.b. cluster 0), fibroblasts/myofibroblasts (n.b. cluster 1), alveolar macrophages (n.b. cluster 2) and plasma cells (n.b. cluster 3) (Fig. 3e), allowing us to study regulatory molecules and signaling within and between clusters. Upstream regulator and pathway enrichment analyses predicted upstream activation in F14^hi^C0 and n.b. cluster 1 of molecules (including TGF-β1, TGF-β2, MRTFB, TEAD1-4 and ISG15) known to be involved in fibrosis (Fig. 3f and Supplementary Table 5). The canonical profibrotic cytokine transforming growth factor beta (TGF-β) (encoded by *TGF-β1*, *TGF-β2* and *TGF-β3*) plays an important role in IPF^25,33^ and has been implicated in ADI cell formation and inhibition of differentiation toward AT1 cells^34^. MRTFB regulates myofibroblast differentiation^35^, while TEAD family members (part of YAP/TAZ coactivator complex) are key effectors of profibrotic pathways including Hippo, TGF-β and WNT signaling^36–38^, implicated in tissue regeneration and fibrosis^26,39^. ISG15, a modulator of p53, has been implicated in age-related signaling pathways^40^. Enrichment of IPF-, glycoprotein VI (GP6)- and wound-healing signaling pathways, along with pathways associated with cell movement and migration, further supports an active fibrogenic node.

Prediction analysis of ligand–target interaction^41^, with directional information preserved (Methods), identified further cell–cell communications within the F14^hi^C0 microenvironment, including TGFB1, IL1B and SFRP2 (Fig. 3g and Supplementary Table 5). *SFRP2* (a WNT signaling modulator) expression distinctly originated from the neighboring fibroblast cluster, implicating potential autocrine and paracrine WNT signaling between (myo)fibroblasts, alveolar epithelial and AbBa cells. The predicted ligand apolipoprotein E (encoded by *APOE*) was highly expressed in the macrophage cluster, alluding to a monocyte-derived and M2-like profile of the neighboring macrophage population^42,43^. By analyzing the expression of all annotated APOE receptors across the F14^hi^C0 region distance, we identified an inverse expression pattern between *APOE* and several of its receptors (Fig. 3h). Glypican 1 (encoded by *GPC1*), LDL receptor-related protein 1 (*LRP1*) and syndecan 1 (*SDC1*) were more highly expressed within, and in close proximity to, the F14^hi^C0 region. These observations suggest a potentially under-recognized role for apolipoprotein signaling within the AbBa cell fibrotic niche in IPF.

### Spatial transcriptomics in a mouse model of pulmonary fibrosis

**Fig. 4:**
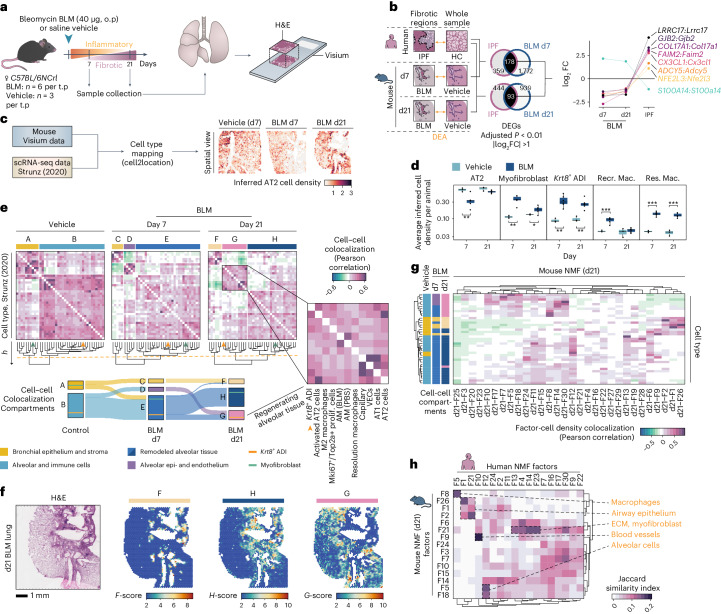
Comparative spatial analysis of pulmonary fibrosis in mouse and human. **a**, Study design for the mouse BLM lung injury model, analyzing lungs collected at days 7 (d7) and 21 (d21) post oropharyngeal (o.p.) administration (*n* = 6 for BLM, *n* = 3 for vehicle per time point (t.p.)), used for Visium experiments. **b**, DEA of fibrotic regions in human IPF and BLM-treated mice versus controls, with Venn diagrams of DEGs unique and common to human IPF and mouse BLM at d7 or d21 and highlighting genes with inverse expression patterns. **c**, Integration of Visium and scRNA-seq data (Strunz et al.^11^; GSE141259) to infer spot cell-type densities, exemplified by inferred AT2 density. **d**, Averaged cell-type abundance per animal, comparing time points and treatments for selected cell types (Welch two sample *t*-test, two-sided; *n*_Vehicle_ = 3 and *n*_BLM_ = 6, per time point). *P < 0.05, **P < 0.01 and ***P < 0.001 (AT2, d7: *P* = 0.0036; myofibroblast, d7: *P* = 0.0011, d21: *P* = 0.0306; *Krt8*^+^ ADI, d7: *P* = 0.0042, d21: *P* = 0.0045; Recr. Mac., d7: *P* = 0.0006; Res. Mac: d7: *P* < 0.0001, d21: *P* < 0.0001). The center line is the median, the box limits are the upper and lower quartiles, the whiskers are 1.5× interquartile range and the points are the value per animal. **e**, Cell–cell correlation heat maps display cellular colocalization compartments (defined by tree height cutoff, *h* = 1.5, orange dashed line) across condition and time point. The Sankey diagram illustrates cell type shifts within compartments from vehicle to BLM d7 to BLM d21, with *Krt8*^+^ ADI (orange line) and myofibroblast (green line) populations highlighted. **f**, Computed compartment scores (F–H) based on cell-type densities for a BLM d21 lung section. **g**, Correlation (Pearson) of BLM d21 NMF factor activity and cell-type densities in all spots. The cell-type group colors match their respective compartments (A–H) from prior analysis. **h**, Comparison between human and mouse d21 NMF analyses using the top 100 factor-driving genes, filtered for factor pairs with a Jaccard index >0.1 to highlight major overlaps. AM, alveolar macrophages; Recr. Mac., recruited macrophages; Res. Mac., resolution macrophages; VECs, vascular endothelial cells; Prolif., proliferating. Source data

**Extended Data Fig. 4:**
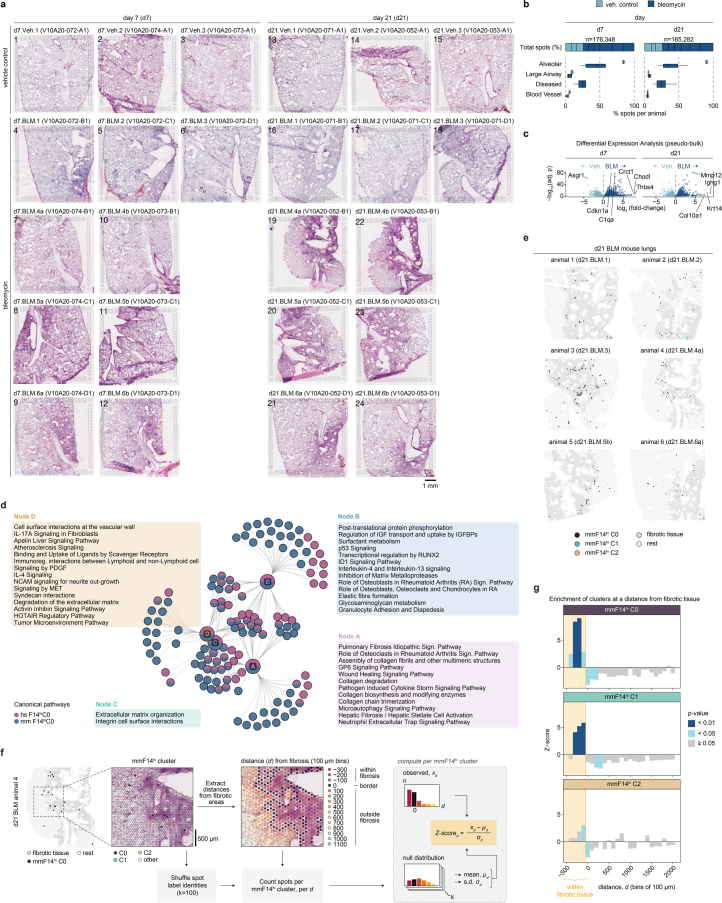
Spatial and molecular analysis of BLM-induced mouse lung fibrosis. **a)** H&E-stained sections of mouse lung tissues collected at day 7 (d7) and day 21 (d21) post-BLM or saline vehicle treatment that were used for Visium analysis. **b)** Summarizing statistics of Visium spot count used and histopathological annotations. Center line, median; box limits, upper and lower quartiles; whiskers, 1.5× interquartile range. **c)** Volcano plots depicting differential expression analysis results between BLM and vehicle-treated lungs at d7 and d21, highlighting genes with significant (adj. p < 0.01) changes in expression. **d)** Network of top significant (p value < 0.0001) canonical pathway enrichment results based on human hsNMF-F14^hi^ C0 and mouse mmNMF_d21_-F14^hi^ C0 marker genes (adj. p < 0.05), with specific pathways within each node cluster annotated. Inner nodes illustrate groups of regulators sharing genetic influences, and outer nodes represent contributing marker genes. **e)** Spatial plots of mmNMF-F14^hi^ C0-C2 and regions histologically annotated as fibrotic tissue (gray) for all the BLM-challenged lungs collected at d21. **f**) To quantify the spatial localization of the mmNMF-F14^hi^ clusters in relation to fibrotic tissue, the same strategy as presented in Extended Data Fig. 2h was employed, except for using regions annotated as fibrotic to extract radial distances. **g)** Z-scores for each cluster plotted across each distance bin, relating to the distance from fibrotic regions (yellow shaded areas) across all tissue samples. P-values were computed from the Z-scores using a two-tailed test (dark blue: p < 0.01; light blue: p < 0.05; gray: ≥ 0.05). mmF14^hi^ C0 was significantly enriched within the border of fibrotic areas, especially at 150-350 µm distances into the fibrotic tissue. On the other hand, there was an underrepresentation of mmF14^hi^ C0 spots in areas outside the fibrosis. H&E, Hematoxylin and eosin. Source data

The BLM mouse model is the most established preclinical model for pulmonary fibrosis^44,45^. To increase understanding of the translational predictivity of this model for human IPF, we generated SRT data from mouse lung samples collected at day 7 (d7) and day 21 (d21) following single-dose BLM or saline (vehicle) administration (Fig. 4a, Extended Data Fig. 4a and Supplementary Table 6). These time points were selected to cover the acute inflammatory and early fibrogenic phase (d7), as well as the established fibrotic stage (d21) (Supplementary Notes 5 and 7).

A total of 151–571 million sequencing reads (average 325 million) per sample were generated (Supplementary Table 6). Healthy alveolar regions accounted for 80–90% and 30–50% of the total number of spots in saline and BLM-challenged lungs, respectively. Remaining spots in the BLM-challenged samples were labeled as areas of tissue damage or remodeling (Extended Data Fig. 4b). A pseudobulk DEA between BLM and vehicle controls identified 3214 and 3787 DEGs at d7 and d21, respectively (Extended Data Fig. 4c).

#### Comparative analysis of gene expression and cellular composition of pulmonary fibrosis in mice

We identified DEGs in annotated fibrotic areas compared with control samples in the mouse model and analyzed their overlap with DEGs in human IPF (Fig. 4b and Supplementary Table 7). Numerous DEGs overlapped between mouse and human (178 between IPF and d7 BLM fibrotic regions and 93 between IPF and d21 BLM), with eight DEGs displaying contrasting fold-change directionality. Among the latter, most are involved in ECM organization (*COL17A1* (ref. ^46^)), inflammatory signaling (*CX3CL1* (ref. ^47^)) and apoptosis regulation and cellular adhesion (*S100A14* (ref. ^48^) and *FAIM2* (ref. ^49^)). While these genes may play a role in fibrosis in both conditions, the inverse expression patterns suggest divergent roles in human IPF and the mouse BLM model.

Cell-type deconvolution was performed using a lung scRNA-seq dataset generated in the BLM mouse model^11^ (referred to as ‘Strunz’^11^). Spatial visualization of cell-type densities demonstrated accurate mapping to relevant tissue regions, where alveolar epithelial cells were inferred in healthy alveolar tissue (Fig. 4c). Pronounced differences in cell-type densities were observed between BLM and vehicle groups, including for resolution (M2-polarized) macrophages and *Krt8*^+^ ADI cells (Fig. 4d). AT2 cell abundance decreased at d7 but showed recovery by d21. The apparent influx of recruited (proinflammatory) macrophages at d7 normalized by d21, confirming resolution of acute inflammation.

#### Dynamic lung tissue remodeling in response to BLM in mice

Colocalization analysis revealed dynamic spatial compartmentalization of cell types within spots, capturing the spatiotemporal dynamics of fibrogenesis and indicating lung tissue remodeling in response to BLM injury (Fig. 4e). In vehicle control lungs, we identified two compartments consisting of bronchial epithelial (A) and alveolar (B) tissue, outlining the uninjured lung architecture. In the d7 BLM-challenged lungs, prominent cell densities consisted of bronchial epithelium (C), alveolar epithelium and alveolar capillary endothelium (D) and remodeled alveolar tissue marked by fibroblasts and myofibroblasts (E). At d21, the cellular composition of the compartments was altered, so that in addition to bronchial epithelium (F) and fibrotic, remodeled alveolar tissue (H), we observed a compartment characterized by alveolar epithelium macrophages and *Krt8*^+^ ADI cells (G), exhibiting a profile of regenerating alveolar tissue (Supplementary Table 8). Spatial mapping confirmed that compartment F aligned with bronchial structures and H coincided with fibrotic/remodeled tissue, while G was present along the borders of fibrotic areas and extending into intact tissue (Fig. 4f).

#### Regenerative signatures reveal distinct epithelial responses in mouse BLM-induced fibrosis

Given that d21 in the mouse model reflected an established stage of fibrosis with minimal acute inflammation, we focused on this time point for comparison with IPF. NMF application to the mouse d21 data (mmNMF_d21_) generated 30 factors (Supplementary Table 9). Factor activity and cell-type abundance colocalization analysis largely reflected the d21 BLM compartmentalization, affirming that NMF effectively captures patterns comparable with the cell-type deconvolution approach (Fig. 4g). The regenerating alveolar epithelial compartment (G) was represented by a set of factors primarily reflecting AT2 cells (F30), alveolar and resolution macrophages (F8) or activated AT2 and *Krt8*^+^ ADI cells (F14). Factors F18, F5 and F7 predominantly represented AT1 and endothelial cells (Supplementary Table 9).

We compared the mouse mmNMF_d21_ factors with the factors identified in the human IPF NMF analysis (hsNMF) (Fig. 4h). The top contributing genes showed overall weak overlap between human and mouse factors. However, factors associated with distinct morphological features, such as smooth muscle cells, blood vessels and ciliated airway epithelium, demonstrated more pronounced overlap, highlighting conserved signatures in normal lung structures, compared with disease or injury responses. Notably, factors containing transcriptional signatures for the human *KRT5*^−^/*KRT17*^+^ AbBa cells (hsNMF-F14) and mouse *Krt8*^+^ ADI cells (mmNMF_d21_-F14) had a limited overlap.

### Translation of the fibrotic microenvironment

#### Contrasting responses in human IPF and mouse BLM model

**Fig. 5:**
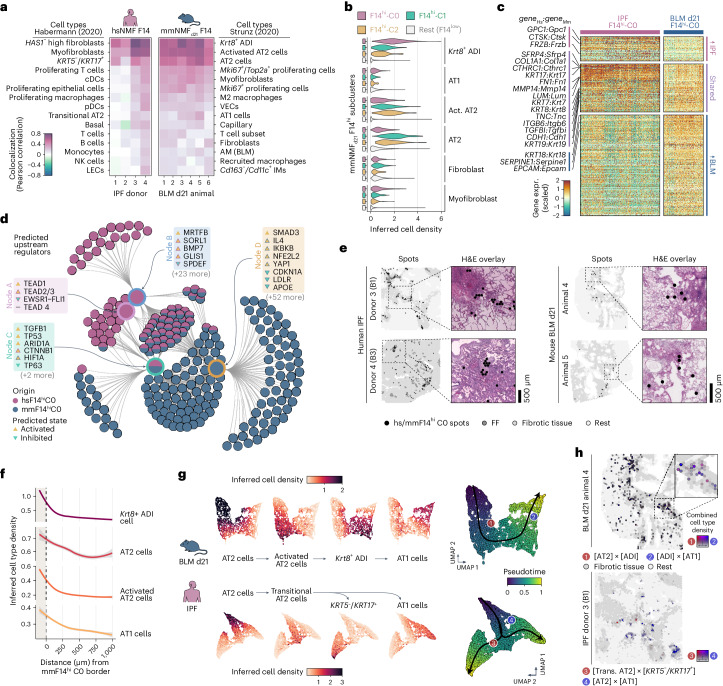
Translational dissection of the fibrotic niche and cellular dynamics. **a**, Correlation of hsNMF-F14^hi^ and mmNMF-F14^hi^ factor activity with the top 15 correlating cell types from Habermann^5^ (human) and Strunz^11^ (mouse) scRNA-seq datasets. **b**, Cell density distribution in mmNMF_d21_-F14^hi^ subclusters showing high abundance of *Krt8*^+^ ADI cells in mmNMF_d21_-F14hi C0 and AT2 cells in C1 and C2. **c**, Heat map with scaled and centered hsNMF-F14^hi^C0 and mmNMF_d21_-F14^hi^C0 marker gene expression (expr.), grouped by higher in IPF, shared or higher in BLM (adjusted *P* < 0.01, avgerage log_2_FC >0). **d**, Comparative network plot of top significant regulators (*P* < 10^−7^, right-tailed Fisher’s exact test) from IPA upstream analyses of marker genes (adjusted *P* < 0.05) in hsNMF-F14^hi^C0 and mmNMF_d21_-F14^hi^C0. The inner nodes illustrate groups of regulators sharing genetic influences and the outer nodes represent contributing marker genes. **e**, Spatial mapping of hsNMF-F14^hi^C0 and mmNMF_d21_-F14^hi^C0 spots within tissue sections illustrating the relationship with fibrotic regions. **f**, Radial distribution line graphs of cell densities around mmNMF_d21_-F14^hi^C0. The gray shading corresponds to the 95% confidence interval. **g**, Spatial trajectories of spots with high inferred densities of AT2, activated AT2, *Krt8+* ADI and AT1 cells (mouse) or AT2, transitional AT2, *KT5-/KRT17*^+^ and AT1 cells (human). **h**, Spatial colocalization of AT2-to-*Krt8*^+^ ADI (1, red) and ADI-to-AT1 (2, blue) inferred cell densities in a mouse lung section and transitional AT2-to-*KRT5*^−^/*KRT17*^*+*^ (3, red) and AT2-to-AT1 (4, blue) densities in one human IPF lung section. Colocalization intensities are visualized on a red–blue spectrum, with mixtures appearing purple and spot opacity reflecting intensity level. Tissue background and areas of fibrosis (gray) are illustrated for context. VECs, vascular endothelial cells; pDC/cDCs, plasmacytoid/classical dendritic cells; NK cells, natural killer cells; AM, alveolar macrophages; IM, interstitial macrophages; Act. AT2, activated AT2 cells; Trans. AT2, transitional AT2 cells. Source data

We analyzed the spatial correlation of the factors containing the *KRT5*^−^/*KRT17*^+^ AbBa cells (hsNMF-F14) in human IPF samples and the *Krt8*^+^ ADI cells (mmNMF_d21_-F14) in mouse BLM samples. hsNMF-F14 activity predominantly correlated with fibroblasts (*HAS1*-hi), myofibroblasts and *KRT5*^−^/*KRT17*^+^ AbBa cells. Conversely, mmNMF_d21_-F14 activity primarily correlated with *Krt8*^+^ ADI cells and AT2 cells, while showing a weaker correlation with myofibroblasts. Additionally, mmNMF_d21_-F14 showed correlation (albeit weaker) with AT1 cells, unlike hsNMF-F14 (Fig. 5a), in line with the distinct fibrogenic environment in the human AbBa niche.

To further compare the AbBa (IPF) and ADI (BLM) niches, we refined mmNMF_d21_-F14 and identified four subclusters (mmNMF_d21_-F14^hi^ C0–3), where cluster 0 (mmNMF_d21_-F14^hi^ C0) exhibited strongest association with *Krt8*^+^ ADI cells (Fig. 5b). We detected shared markers between hsNMF-F14^hi^ C0 and mmNMF_d21_-F14^hi^ C0, including several collagens and ECM-related genes (for example, *COL1A1*, *FN1*, *TNC* and *CTHRC1*), epithelial cell markers (*CDH1*) and markers for human AbBa cells (*KRT17*) and mouse ADI cells (*KRT8*) (Fig. 5c and Supplementary Table 10). This suggests shared traits between AbBa and ADI regions involving ECM remodeling and a basaloid phenotype, which was further supported by pathway analysis (Extended Data Fig. 4d). However, the ADI-related gene signature predominantly engaged pathways related to inflammation and repair, whereas the AbBA signature reflected the chronic and progressive nature of IPF, dominated by immune responses and pathways governing long-term tissue remodeling.

For better understanding of the aberrant fibrotic niche drivers, we performed an upstream regulator analysis for hsNMF-F14^hi^ C0 and mmNMF_d21_-F14^hi^ C0 (Fig. 5d and Supplementary Table 11). Both groups had predicted activation of TGFB1, TP53 and SMAD3, suggesting a conserved TGF-β-related mechanism^25,50,51^ and cellular senescence^52^. hsNMF-F14^hi^ C0-specific regulators included the antifibrotic growth factor BMP7, APOE receptor SORL1 and GLIS1, a component of the Notch signaling pathway. mmNMF_d21_-F14^hi^ C0 showed activation of oxidative stress and inflammation regulators including HIF1A, IL4, YAP1 and NFE2L2 (NRF2). Contrary to our findings in the human samples suggesting an activating role for apolipoprotein signaling upon hsNMF-F14^hi^ C0 (Fig. 3f–h), APOE and its receptor LDLR were predicted to be inhibited for mmNMF_d21_-F14^hi^ C0 in the mouse samples.

Next, we examined the histological context of the mmNMF_d21_-F14^hi^ clusters and quantified their presence in relation to fibrosis (Fig. 5e and Extended Data Fig. 4e–g). The mmNMF_d21_-F14^hi^ C0 cluster was found within fibrotic regions, often near the junction between healthy and fibrotic tissue, comparable with the spatial relation of FF and hsNMF-F14^hi^ C0 in the IPF samples and suggesting a transitional state.

The BLM *Krt8*^+^ ADI transitional cell population is predicted to originate from either AT2 cells or club cells and differentiate into AT1 cells^11^. By assessing the cell-type densities in relation to radial distance from the borders of the mmNMF_d21_-F14^hi^ C0 niche, we identified high densities of AT2, activated AT2 and AT1 cells close to the niche (Fig. 5f). These observations shared similarities with the corresponding IPF hsNMF-F14^hi^ C0 analysis (Fig. 3c), highlighting the absence of AT1 cells around the human AbBa niche.

In BLM mouse data, spatial trajectory analysis of the alveolar epithelial cell types/states (Fig. 5g) showed a single path from AT2 cells through activated AT2 and ADI cells to AT1 cells. Conversely, in IPF lungs, the trajectory branched from AT2 cells through transitional AT2 cells and diverged into either *KRT5*^−^/*KRT17*^+^ AbBa or AT1 cells. Spatial visualization of the trajectories further illustrated the continuous colocalization of these cell types in BLM-injured mouse lungs and spatial separation of transitional AT2–AbBa and AT2–AT1 niches in IPF (Fig. 5h).

#### Immune cell dynamics in pulmonary fibrosis

**Extended Data Fig. 5:**
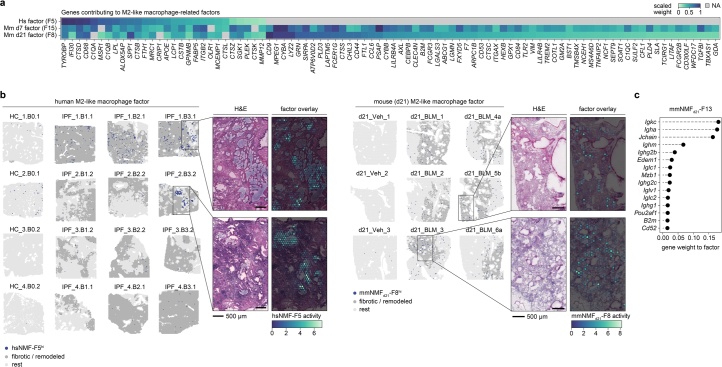
Profibrotic macrophages and immune cell profiles. **a)** Heatmap showing the gene contributions to the identified M2-like macrophage-related factors across human lungs (hsNMF-F5) and mouse lungs at day 7 (d7) (mmNMF_d7_-F15) and day 21 (d21) (mmNMF_d21_-F8). The visualized genes are those, among the top 100 contributing genes, that overlapped between at least two of the three groups. Color represents each gene’s factor contribution as their scaled weight, where 1 is assigned to the strongest contributing gene to the factor out of all genes in the data set. **b)** Spatial distribution of macrophage-associated factors in human (hsNMF-F5) and mouse d21 (mmNMF_d21_-F8) tissue sections. The top 99^th^ percentile of either factor was labelled as hsNMF-F5^hi^ or mmNMF_d21_-F8^hi^ and visualized together with the histopathological spot annotations grouped into ‘fibrotic / remodeled’ tissue (gray) and remaining tissue (light gray). Zoomed in graphs display factor activity overlaid the H&E images for selected regions that demonstrated high factor activity in areas of bronchial epithelium within fibrotic tissue. **c)** Top 15 contributing genes to mmNMF_d21_-F13, which was identified to contain a strong plasma cell profile driven by *Igha*, among other immunoglobulin-related genes. H&E, Hematoxylin and eosin. Source data

Our NMF analysis revealed factors in the IPF and the d7 and d21 BLM datasets that shared key marker genes indicative of macrophages (*SPP1*, *CD68* and *APOE*) (Extended Data Fig. 5a). *SPP1*^+^ profibrotic macrophages, displaying an M2-polarization phenotype, have previously been implicated in ECM remodeling and fibrosis^6,53^. In the two IPF donor lungs containing bronchial honeycombing, the macrophage factor (hsNMF-F5) activity was particularly pronounced within these regions (Extended Data Fig. 5b). The hsNMF-F5 activity varied across samples and donors, potentially owing to tissue heterogeneity or constraints from the chosen number of NMF factors.

**Fig. 6:**
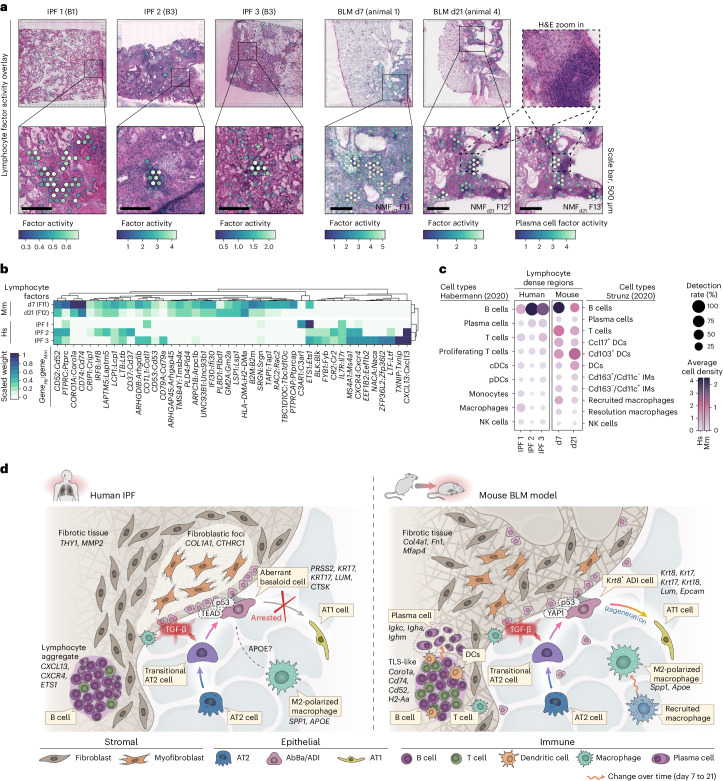
Immune cell signatures and comparative overview of the fibrotic niche in IPF and the BLM mouse model. **a**, Spatial visualization of NMF factors overlapping dense lymphocyte or immune cell aggregates in selected human and mouse samples. Scale bars, 500 µm. Imaged at 20× magnification. **b**, Heat map displays the top contributing factor genes across condition, filtered to show genes with a summed scaled weight above 0.5 across the groups. **c**, Dot plot with inferred cell-type densities, for selected immune cell types from the ‘Habermann^5^’ and ‘Strunz^11^’ datasets, in the most active spots of the selected human and mouse factors. **d**, Schematic summary of the fibrotic niche in human IPF lungs and in mouse BLM-injured lungs, illustrating the proposed cellular interplay within the fibrotic lungs. A key distinction between IPF and the BLM mouse model was centered around the diverging regenerative properties of the IPF-associated AbBa cells versus the mouse Krt8^+^ ADI cells. While both populations exhibit signs of senescence (p53), the mouse ADI state appears to maintain a functional balance that still prompts it to differentiate into AT1 cells. TGF-β and Wnt-related (TEAD, YAP1) signaling pathways were central within the fibrotic niche, and the presence of immune cells in proximity to, or within, the severely remodeled tissue implies active fibrogenic modulatory roles. Pro-fibrotic M2-polarized (‘resolution’) macrophages with similar gene signatures, expressing *SPP1* (*Spp1*) and *APOE* (*Apoe*) were detected in both human IPF and mouse BLM-injured lungs. In contrast to human IPF AbBa regions, a predicted negative APOE upstream signaling was identified in mouse ADI regions. In mouse, the recruited proinflammatory macrophages seen at the early time point post BLM-installation were absent by d21. Establishment of plasma cells adjacent to TLS-like areas in the BLM-injured mice occurred at the later time point. pDC/cDCs, plasmacytoid/classical dendritic cells; IMs, interstitial macrophages; NK cells, natural killer cells; TLS, tertiary lymphoid structure. Source data

A shared histological feature between the IPF and BLM-injured lungs was presence of dense immune cell infiltrates embedded within the fibrotic tissue (Fig. 6a). In the time-point-separated mouse NMF analyses, mmNMF_d7_ and mmNMF_d21_ (Supplementary Table 9), we identified factors prevalent in these regions. In the human NMF, a similar factor was not consistently detected. Therefore, more targeted donor-specific NMF analyses were performed, which identified factors in three of the donors (IPF 1–3) overlapping spatially with the observed immune infiltrates. Gene expression (Fig. 6b) and cell-type composition (Fig. 6c) within the immune-dense regions revealed notable differences between IPF and BLM lungs. In the BLM model, enrichment of genes such as *Cd74* and *Coro1a* indicated presence of antigen-presenting cells and lymphocytes^54^. Additionally, a distinct plasma cell factor, driven by IgA heavy chain (*Igha*) expression (Extended Data Fig. 5c), was identified in proximity to the d21 BLM immune-dense structures (Fig. 6a). Overall, the BLM regions demonstrated a relatively balanced mixture of B, T and dendritic cells, in contrast to the human IPF samples, where a pronounced expression of *CXCL13* suggested a B cell-dominated immune response^55^, which was further supported by the cell-type composition data. Given recent descriptions of their presence in both healthy and diseased lungs^56^, lymphocytes and plasma cells may modulate fibrosis progression.

Taken together, our analyses delineate distinct cellular trajectories and molecular mechanisms in the fibrotic niche of human IPF and the BLM mouse model (Fig. 6d). We highlight the arrested alveolar cell regeneration in IPF lungs versus the active repair in the BLM model, alongside distinct signaling molecules, such as TGF-β, APOE, YAP1 and TEAD, and differences in immune cell presence. While we recognize the complexity and heterogeneity of IPF and aspects that remain unexplored (Supplementary Note 6), these comparative insights underscore the unique characteristics of fibrosis in human IPF.

## Discussion

Our study presents comprehensive comparative genome-wide spatial transcriptome maps of the diverse cellular ecosystems and distinct molecular signatures in the human IPF lung and the BLM mouse model. Investigations into prefibrotic alveolar tissue and the leading edge of fibrotic remodeling offer potential for identification of targetable early disease-associated molecules for therapeutic intervention.

We propose a central involvement of TGF-β signaling in IPF, alongside other mediators such as TP53, SMAD3, BMP7, MRTFB, TEAD, GLIS1 and APOE, which are linked to senescence, myofibroblast activation and differentiation, Notch and Wnt signaling, apoptosis and cell migration.

Using data factorization, we identify the *KRT5*^−^/*KRT17*^+^ AbBa population in IPF and the *Krt8*^+^ ADI population in the BLM model. Analyzing their proximate neighborhoods delineated critical regions within the fibrotic landscape, and the complex signaling networks identified within these niches suggest these cells participate in the fibrotic response through paracrine signaling and local microenvironment modulation. TGF-β1, a profibrotic cytokine with a major role in IPF pathogenesis^25,33^ and SMAD3, integral to the TGF-β signaling pathway^57^, were predicted as upstream regulators in human AbBa and mouse ADI fibrotic niches, pointing to a shared TGF-β-driven fibrotic signaling pathway. Furthermore, our data suggest that the role of APOE signaling within the IPF fibrotic niche is more substantial than previously appreciated. This warrants further exploration into the potential regulatory function of APOE in IPF, given its well-documented function in lipid metabolism and its emerging role in immunomodulation and fibrosis^58,59^.

Through tracing the alveolar epithelial cell spatial trajectory, we observed an AT2–ADI–AT1 lineage in the mouse model that is preserved in situ, supporting previous single-cell and in vitro studies^11,60,61^ and indicating an ongoing postinjury repair mechanism. In contrast, human IPF lungs depicted a divergent path, with AT2 cells branching into either AbBa cells or AT1 cells. The apparent disruption in the IPF lung regenerative process is in line with descriptions of AbBa cell persistence as an intermediate, nonregenerative state^5^, potentially driving the progressive, irreversible fibrosis in IPF, as opposed to the resolution of fibrosis following acute injury in the mouse model. These findings highlight key challenges in translating animal models to human disease and suggest that the acute BLM mouse model might offer valuable insight into alveolar regeneration. Application of SRT to other pulmonary fibrosis animal models, such as the repeat BLM instillation model^62^ in which a more persistent, senescent *Krt8*^+^ transitional alveolar cell state has been identified^52^, could potentially provide more insights into disease progression in the IPF lung.

Taken together, our study illustrates the potential for spatial transcriptomics to deepen our understanding of IPF pathology and offers rich datasets to further probe the complex cellular interplay in pulmonary fibrosis. This work provides resolution of key mechanisms underpinning IPF and proposes a divergent cellular trajectory toward arrested regeneration in the human IPF lung, as a potential target for the discovery of innovative disease modifying therapies.

## Methods

### Ethical considerations

Human IPF lung samples were acquired with approval by the Human Research Ethics Committee in Gothenburg, Sweden (permit number 1026-15) and HC lung samples were acquired with approval by the Human Research Ethics Committee in Lund, Sweden (permit number Dnr 2016/317). All participants gave written informed consent before inclusion and did not receive any compensation for their participation.

All mouse experiments were approved by the Gothenburg Ethics Committee for Experimental Animals in Sweden and conformed to Directive 2010/63/EU. The present study was approved by the Research Ethics Committee in Gothenburg, Sweden (permit number 31-5373/11). Animal handling conformed to standards established by the Council of Europe ETS123 AppA, the Helsinki Convention for the Use and Care of Animals, Swedish legislation and AstraZeneca global internal standards.

All investigations were performed in accordance with the declaration of Helsinki.

### Experimental methods

#### Human lung tissues

IPF lung tissue was obtained from patients that had undergone lung transplant. Healthy lung tissue was obtained from deceased donors without known lung disease. Human tissues were collected by resection from peripheral lung. Fresh-frozen tissues were obtained from four HC subjects and four patients with IPF. For each patient with IPF, three different tissues were selected to represent areas of mild (‘B1’), moderate (‘B2’) or severe (‘B3’) fibrosis within the same donor, as determined by histological inspection (see Supplementary Table 1 for additional details regarding donors and samples).

#### Mice and BLM challenge

Female C57BL/6NCrl mice (Charles River, Germany) were 8 weeks old on the day of arrival. After a 5-day acclimatization period, the mice were challenged with 30 μl saline or BLM (Apollo Scientific, BI3543, Chemtronica Sweden; 40 µg per mouse) dissolved in saline via oropharyngeal route administration. Lungs were collected at d7 (*n* = 6 for BLM, *n* = 3 for vehicle) or d21 (*n* = 6 for BLM, *n* = 3 for vehicle) following administration.

The mice were housed in Macrolon III cages with poplar chips (Rettenmeier and Sohne), shredded paper, gnaw sticks and a paper house in 12 h–12 h light–dark cycle at 21 ± 1 °C, 55 ± 15% relative humidity with free access to food (R70, Lantmannen AB) and tap water.

#### Mouse tissue collection

Mice were anesthetized with isoflurane (5%, air flow ~2 l min^−1^, maintained at 3% with ~0.7 l min^−1^ air flow). An incision was made from the stomach up to the chin. Heparin (0.1 ml) was injected intracardially through the diaphragm, and the abdominal aorta and apex of the heart was cut. Pulmonary circulation was perfused with 0.8 ml 37 °C saline followed by 0.6 ml 37 °C low-temperature melt agarose (SeaPlaque). The left lung lobe was inflated with 0.4–0.5 ml 37 °C low-melt agarose via the trachea and tied off, collected, snap frozen in prechilled NaCl over dry ice and stored at −80 °C.

The methods describing animal monitoring and sample collection and analyses for pre-study evaluation of BLM time points are outlined in Supplementary Note 7.

#### Generation of SRT

Optimal cutting temperature (OCT)-embedded human lung tissue blocks and agarose-inflated mouse lungs (left lobe) were cryosectioned at 10 µm (mouse) or 12 µm (human) with cryostat temperatures of −20 °C. RNA quality for human samples were assessed by extracting RNA from ten sections using the RNeasy Plus Mini kit (Qiagen) and estimating RNA integrity numbers (RIN) with a 2100 Bioanalyzer (Agilent), which ranged from 5.4 to >8, except for one sample with a RIN of 3 (IPF donor 2, B3). Despite lower RINs for some samples, tissues with satisfactory histological integrity were included in further analyses. For mouse sections, RNA was extracted similarly with the RNeasy micro kit (Qiagen). The RIN values were estimated to >9 for all samples using a 5300 Fragment Analyzer (Agilent).

The tissue sections were placed on Visium Gene Expression slides and stored at −80 °C until further processing. The methanol fixation, hematoxylin and eosin (H&E) staining and imaging Visium protocol (10x Genomics) were followed. Human lung sections were imaged using Axio Imager.Z2 (ZEISS) light microscope at 20× magnification and stitched using Vslide (MetaSystems). Mouse lung sections were imaged at 20× magnification using an Aperio Digital Pathology Slide Scanner (Leica Biosystems).

The sequencing libraries were prepared using the Visium Spatial Gene Expression Slide and Reagent Kit (cat. no. 1000184, 10x Genomics) with the Dual Index Kit TT Set A (cat. no. 1000215, 10x Genomics) according to the Visium Spatial Gene Expression User Guide (Rev C). The human and mouse libraries were pooled separately and sequenced on a NovaSeq 6000 (Illumina) with an S4 flowcell.

#### Histopathology annotations

Histopathological assessments were performed on H&E-stained tissue sections using the Loupe Browser (10x Genomics) software and were manually annotated based on tissue morphology. The human lung data were classified into ‘blood vessel’, ‘large airway’, ‘diseased (remodeled) tissue’, ‘fibroblastic foci/fibrous tissue’, ‘inflammation’ and ‘within normal limits’ (alveolar), where ‘inflammation’ was distinguished as areas with aggregations of immune cells and ‘diseased tissue’ corresponding to clearly recognizable changes in normal lung architecture. The ‘fibroblastic foci/fibrous tissue’ was annotated based on microscopic features, including nuclear density and shape, and increased collagenous matrix. Since this selection also encompassed fibrous tissue that did not classify as FF, a second layer of annotations were performed to specifically identify the FF. Mouse data were annotated as ‘blood vessel’, ‘large airway’, ‘within normal limits’ (alveolar), ‘inflammation (d7)’, ‘inflammation (d21)’ and ‘suspect fibrosis/fibroplasia (d21)’. The ‘inflammation (d7)’ areas comprised an indistinguishable mix of inflammatory and fibrotic tissue, while ‘inflammation (d21)’ labeled dense immune cell aggregates. Consequently, both spots labeled as ‘inflammation (d7)’ and ‘suspect fibrosis/fibroplasia (d21)’ contains fibrotic tissue.

### Computational processing and analysis

#### Processing of Visium sequencing data

FastQ files were processed using the Space Ranger 1.2.2 (10x Genomics) pipeline. Sequence reads were mapped to the respective reference genomes GRCh38 (human) and mm10 (mouse). Manual alignment to the fiducial frame and identification of tissue-covered spots was performed using Loupe Browser (v.6, 10x Genomics).

#### Spatial cell-type mapping

For human samples, spatial deconvolution was performed using cell2location^28^ (v. 0.1) against a previously published scRNA-seq dataset (Gene Expression Omnibus (GEO) accession GSE135893)^5^. The method uses signatures from the provided scRNA-seq data to infer absolute numbers (density) of cell types within each spatial spot. The single-cell regression model was trained with max_epochs = 250 after selecting genes with parameters nonz_mean_cutoff = 1.25, cell_count_cutoff = 5 and cell_percent_cutoff = 0.05. The cell2location model was thereafter obtained with parameters max_epochs = 10,000, detection_alpha = 20 and *n* = 7. For the mouse data, a scRNA-seq dataset from the BLM mouse model (collected at multiple time points including d7 and d21) was used (GEO accession GSE141259)^11^. max_epochs = 400 was set for single-cell model generation using the parameters nonz_mean_cutoff = 1.10, cell_count_cutoff = 4 and cell_percent_cutoff = 0.02 for gene selection. For model training, max_epochs = 15000, detection_alpha = 20 and *n* = 7 was applied.

#### Downstream data quality control and processing

Filtering, processing and analyses of the Visium data were performed in R (v.4.0.5) using the STUtility (v.1.1.1)^63^, Seurat (v.4.1.1)^64^ and ‘semla’^65^ (v. 1.1.6, under R v. 4.2.3 and Seurat v. 4.3.0.1) packages.

For the human IPF and HC samples, tissue-covered spots were selected, and data were imported into R via the STUtility function ‘InputFromTable’. Initial filtering settings were: minimum 350 UMIs per spot, 100 UMIs per gene, 10 genes per spot and five spots per gene. Additional filtering excluded spots with more than 30% mitochondria and/or hemoglobin gene expression. Gene information was retrieved via biomaRt (ref. ^66^) and used to select for ‘protein coding’, ‘IG’ (immunoglobulin) and ‘TR’ (T cell receptor) gene biotypes, as well as to flag X and Y chromosome genes for removal to avoid sex biases. Normalization and scaling was performed with ‘SCTransform’^67^ (Seurat package), specifying sample ID and donor as variables to regress out, to remove major effects of technical and interindividual differences.

The mouse data were filtered in a similar manner, apart from omitting the number of genes per spot (‘minGenesPerSpot’) cutoff, and filtering for number of UMIs per spot was set to 300. ‘SCTransform’ was used for normalization, specifying animal ID as a variable to regress out.

All thresholds for filtering were set based on initial examination of the raw data to exclude low quality spots (or spots outside of tissue areas) and genes with low expression.

#### Visium pseudobulk DEA

For DEA between conditions, pseudobulk datasets were generated by aggregating raw counts per gene across all spots belonging to a donor or animal. DESeq2 (ref. ^68^) (v. 1.30.1) was used for differential gene testing by specifying ‘condition’, with ‘control’ as reference in the design.

For comparison of the normal-appearing alveolar and the fibrotic tissue in IPF samples, gene count data were aggregated into pseudobulk based on histological annotations of ‘within normal limits (alveolar)’, ‘diseased (remodeled) tissue’ and ‘fibroblastic foci/fibrous tissue’. These regions were compared against HC alveolar regions using DESeq2 in two analyses (HC—alveolar versus IPF—alveolar and HC—alveolar versus IPF—fibrotic). Overlapping upregulated DEGs (*n* = 223; adjusted *P* value <0.01, log_2_ fold change (log_2_FC) >0) between the two analyses were used to compute Pearson correlation between expression values in alveolar tissue and distance (0–500 µm) from the fibrotic region border. This analysis was performed on all IPF samples with available histological annotation (*n* = 12). Radial distances from fibrotic areas were calculated using the ‘semla::RadialDistance’ function with ‘remove_singletons=FALSE’, after which only data from fibrotic and alveolar regions were kept before computing gene expression versus distance correlations. The *P* values were adjusted using the Benjamini–Hochberg method, and expression of significantly (adjusted *P* < 0.05) correlated genes were visualized along the fibrosis–distance axis (distances −1,000 to 1,000 µm) as line plots using a generalized additive model (GAM) for smoothing.

For translational comparison of fibrotic regions between IPF and BLM d7 or d21, pseudobulk data were generated by pooling counts on donor/animal level from spots labeled as fibrotic, FF, remodeled or inflamed (BML d7) in disease condition samples. These data were compared with control samples using DESeq2, with ‘condition’ set as design, for each species and time point. Orthogene^69^ (v. 1.4.2) was used to identify mouse orthologues of human genes, and the results were filtered to include only genes with available orthologues and present in all datasets (total of 12,611 genes) (Supplementary Table 7).

#### NMF

Deconvolution through NMF was applied to the Visium gene expression data using the ‘RunNMF’ function in STUtility, which relies on the NNLM package (v. 0.4.4). The method decomposes data into a set number of factors that are expressed as non-negative values (activity) within each data point (spot) along with a feature (gene) loading matrix, describing the contribution (weight) of each gene to the factors. The full human (HC and IPF) dataset was deconvolved into 30 factors (‘hsNMF’), while the mouse data (vehicle control and BLM) were split by time point (d7, d21) before each subset was deconvolved into 30 factors (mmNMF_d7_, mmNMF_d21_). To describe each factor, functional enrichment analysis of the top 25 most contributing genes for each factor was performed using the ‘gost’ function in the gprofiler2 (v. 0.2.1) R package^70^, with the ‘hsapiens’ (human) or ‘mmusculus’ (mouse) organism specified. All factors were further annotated by examining the top contributing genes, the spatial localization of factor activity and their abundance in different samples (diseased or control).

To compare factors across species, the Jaccard similarity index was computed using the top 100 contributing genes for each factor, calculated as the intersection over the union of each gene set pair.

The distribution of each factor within the human samples (hsNMF) was assessed by calculating the frequency of spots in the 99th percentile of factor activity (F^hi^) versus the total number of spots in each tissue block category (B0–3). Spatial colocalization of factors and cell types was analyzed using pairwise Pearson correlation between spot factor activity and inferred cell-type density. To explore donor variability in colocalization, the human data were grouped into HC (all HC donors), IPF donor 1, IPF donor 2, IPF donor 3 and IPF donor 4, before correlation analysis.

For subclustering of hsNMF-F14, a principal component (PC) analysis was performed on the most active (99th quantile) spots (F14^hi^) and PCs 1–8 were used as input for ‘FindNeighbors’ and ‘FindClusters’ (resolution of 0.4), which generated five clusters. The mmNMF_d21_ F14^hi^ spots were subclustered using the same approach but with PCs 1–14 as input and clustering resolution of 0.5, obtaining three clusters.

#### Spatial enrichment in fibrotic regions

For IPF samples, we used the histopathological annotations to extract the radial distances, *d*, from the areas of FF, using the ‘RadialDistance’ function in ‘semla’^65^ (v. 1.1.6; R v. 4.2.3; Seurat v. 4.3.0.1) with kept singletons. Distances of 0 µm represent the outer line of spots immediately exterior to the FF-labeled spots. Distances were binned into groups of 100 µm (corresponding to one row of spots), and the F14^hi^ spot count was registered in each bin. To produce a null distribution, the F14^hi^ cluster labels were shuffled across all spots within each sample, *k* = 100 times. Distance bins >3,000 µm were discarded to exclude distant tissue areas. For each cluster, the observed count at every bin (*x*_d_), the mean (*µ*_d_) and the standard deviation (*σ*_d_) across all randomization rounds were used to compute a *Z*-score per distance bin as: ${Z}_{{\mathrm{d}}}=\frac{{x}_{{\mathrm{d}}}-{\mu }_{{\mathrm{d}}}}{{\sigma }_{{\mathrm{d}}}}$. The same approach was applied to the d21 BLM mouse data to analyze spatial enrichment of the mmNMF_d21_ F14^hi^ clusters in relation to areas of fibrosis (annotated ‘suspect fibrosis/fibroplasia’). The *P* values were computed from absolute *Z*-scores using a two-tailed *t*-test.

#### Expression radial distance analysis from F14^hi^ clusters

Fluctuations in gene expression and cell-type densities along a radial distance from the hsNMF-F14^hi^C0 or mmNMF_d21_ F14^hi^C0 regions (region of interest, ROI) were computed. Distance information from all tissues containing a ROI was extracted using ‘semla’^65^ (v. 1.1.6; R v. 4.2.3; Seurat v. 4.3.0.1) with the ‘RadialDistance’ function, where singletons were excluded in the human analysis. For human IPF data, distance correlation coefficients (Pearson) were computed for the 1,000 most variable genes within 500 µm from the ROI. *P* values were corrected using the Benjamini–Hochberg method. The cell-type density correlations were similarly analyzed. The cell density and gene expression fluctuations were visualized using the ‘geom_smooth’ function (ggplot2, v. 3.4.0) with GAM and formula ‘y ~ s(x, bs = ‘cs’)’. For mouse BLM d21 data, ‘geom_smooth’ with the locally estimated scatterplot smoothing method (‘loess’) was used for visualization of cell density data.

#### IPF fibrotic niche regulators and cell–cell communication

To investigate the microenvironment surrounding hsNMF-F14^hi^C0 spots, the neartest n.b. (≤2 spot distance) were identified using the ‘RegionNeighbours’ STUtility function. The neighboring spots were clustered using PCA (PCs 1–9) through ‘FindNeighbors’ and ‘FindClusters’ (resolution of 0.2), resulting in six clusters (n.b. clusters 0–5). The marker genes were determined using ‘FindAllMarkers’, comparing each cluster to all others. Due to their low abundancies, n.b. clusters 3–5 were omitted from some downstream analyses.

Upstream regulators and active pathways for n.b. clusters (0–2) were predicted with Ingenuity Pathway Analysis (IPA; version 90348151, Ingenuity Systems, Qiagen) based on marker genes (adjusted *P* < 0.01). The marker genes for hsNMF-F14^hi^C0, generated by comparing hsNMF-F14^hi^C0 spots against all others in the IPF Visium subset using ‘FindMarkers’ (‘min.pct = 0.25’ and ‘min.diff.pct = 0.1’), were also analyzed. Results were assessed with multienrichjam (v. 0.0.72.900)^71^, visualizing the top 20 upstream regulators and top ten pathways and diseases/functions.

Directional cell–cell communication between n.b. clusters was analyzed using NicheNet (v. 1.1.1)^41^. This method predicts ligand–target links from gene expression and incorporates intracellular signaling. Ligand–receptor interactions (‘lr_network.rds’), ligand–target gene regulatory potential scores (‘ligand_target_matrix.rds’) and weighted ligand-signaling and gene regulatory networks (‘weighted_networks.rds’) were retrieved from the NicheNet data repository (10.5281/zenodo.3260758). Analyses were conducted in four rounds, alternating receiver and sender populations: (1) F14^hi^C0 with n.b. clusters 0–5 as senders, (2) n.b. cluster 0 with F14^hi^C0 as sender, (3) n.b. cluster 1 withF14^hi^C0 as sender and (4) n.b. cluster 2 withF14^hi^C0 as sender. In each round, receiver genes were identified using data from spots not included in the analysis (of IPF and HC origin). The results across all rounds were compiled, and the top prioritized ligands (average correlation >0.075) from round 1 were used to visualize corresponding results in subsequent rounds and across all relevant clusters.

#### Spatial cell-type compartmentalization in mouse

Cell-type colocalization compartments were identified in mouse Visium data using the Strunz (2020) cell2location results. Cell types labeled as ‘NA’ and ‘low.quality.cells’ were excluded. Visium spot data were grouped into vehicle (d7 and d21), BLM d7 and BLM d21 subsets, and pairwise correlations (Pearson) were computed for each cell type across all spots within each group. Hierarchical clustering defined compartments with a tree height (*h*) cutoff of 1.5. A Sankey diagram visualized cell types in each compartment for each subset. For BLM d21 compartments F, H and G, spot-wise compartment scores were calculated by summing the inferred densities for all cell types within each compartment.

#### Translational analyses of AbBa clusters

Samples IPF 3 B1–B3, IPF 4 B1–B3 and BLM d21 animals 1–5 were selected for analysis, based on their pronounced fibrosis and AbBa cell-dense regions. The raw counts were filtered to include only orthologous genes, identified by orthogene^69^. A new assay was created from the filtered data for separate normalization of human and mouse datasets, followed by integration using Seurat’s ‘FindIntegrationAnchors’ and ‘IntegrateData’. Anchor features were selected by ‘SelectIntegrationFeatures’. The marker genes for hsNMF-F14^hi^C0 and mmNMF_d21_ F14^hi^C0 were separately identified using ‘FindMarkers’, comparing against all other same-species spots, using the integrated genes (Supplementary Table 10).

Marker genes (Bonferroni adjusted *P* < 0.05) were analyzed for upstream regulator and canonical pathway enrichment using IPA. Comparisons across species were conducted with multienrichjam (ref. ^71^) (v.0.0.72.900) to identify shared and unique regulators and pathways. The most significant findings (*P* < 10^−7^ for regulators and *P* < 10^−4^ for pathways, right-tailed Fisher’s exact test) were visualized in clustered network (cnet) plots, grouping predicted molecules in clusters (‘Nodes’) based on shared marker genes.

#### Lymphocyte aggregate comparison

To identify gene signatures overlapping histological findings of lymphocyte aggregates, the human IPF data was split by donor and individually processed using ‘SCTransform’ and NMF, to produce 30 new subject-specific factors for each donor (hsNMF_IPF_). For the mouse data, the NMF results produced for each time point was used (mmNMF_d7_ and mmNMF_d21_).

Activities of the top 100 contributing genes for each factor were scaled between 0-1. Cell densities and detection rates were estimated for spots within the 99th percentile of factor activity. Some mouse cell types that lacked human comparatives or relevant signals were excluded from visualization (‘AM (BLM)’, ‘AM (PBS)’, ‘non classical monocytes (*Ly6c2*-)’, ‘*Fn1*^+^ macrophages’, ‘M2 macrophages’, ‘Themis T cells’ and ‘T cell subset’). Average cell densities and detection rates in spots with density score >0.5 were calculated and visualized.

#### Spatial cell colocalization trajectory analysis

Inferred cell-type densities of ‘AT2 cells’, ‘activated AT2 cells’, ‘*Krt8*^+^ ADI’ and ‘AT1 cells’ (mouse) or ‘AT2’, ‘Transitional AT2’, ‘*KRT5*^−^/*KRT17*^+^’ and ‘AT1’ (human) were used to select spots in the 95th percentile of abundance for uniform manifold approximation and projection (UMAP) dimensionality reduction (n.neighbors = 30, min.dist = 0.1) and to generate low-resolution clusters with ‘FindNeighbors’ and ‘FindClusters’ (mouse: resolution of 0.2, human: resolution of 0.1), identifying clusters corresponding to AT2-dense spots (‘AT2-cluster’). Slingshot^72^ (v. 1.8) trajectory analyses were applied to each UMAP embedding with ‘getLineages’ assigning AT2-clusters as starting points. The curves were extrapolated using ‘getCurves’ (approx_points = 300, thresh = 0.01, stretch = 0.8, allow.breaks = FALSE, shrink = 0.99). Pseudotime was estimated by integrating gene counts (detected (greater than five transcripts) in ≥1% of spots) and Slingshot curves into a negative binomial GAM using ‘fitGAM’ (tradeSeq, v. 1.4.0)^73^. Two curves were identified in the human data, and the visualized pseudotime represents the maximum values of the two curves.

### Statistics and reproducibility

Statistical methods for each analysis are detailed in their respective method sections. No statistical method was used to predetermine sample size. For human samples, it was maximized based on availability, tissue quality, cost and throughput, and the number of donors aligns with similar spatial transcriptomics studies^20,56,74^. The number of BLM-challenged mice exceeded that of controls to account for possible study dropouts due to adverse effects; however, all mice met the inclusion criteria and were retained. Technical replicates were included for most human and mouse samples and produced consistent results, ensuring reproducibility. Investigators were not blinded to disease status or treatment during experiments and analyses.

### Reporting summary

Further information on research design is available in the Nature Portfolio Reporting Summary linked to this article.

## Online content

Any methods, additional references, Nature Portfolio reporting summaries, source data, extended data, supplementary information, acknowledgements, peer review information; details of author contributions and competing interests; and statements of data and code availability are available at 10.1038/s41588-024-01819-2.

### Supplementary information

Supplementary Information Supplementary Notes 1–7, Figs. 1–3, references and legends.

Reporting Summary

Supplementary Tables Supplementary Tables 1–11.

Supplementary Data Source data Supplementary Fig. 3.

### Source data

Statistical Source Data Statistical source data for Fig. 1.

Source Data Fig. 2 Statistical source data for Fig. 2.

Source Data Fig. 3 Statistical source data for Fig. 3.

Source Data Fig. 4 Statistical source data for Fig. 4.

Source Data Fig. 5 Statistical source data for Fig. 5.

Source Data Fig. 6 Statistical source data for Fig. 6.

Source Data Extended Data Fig. 2 and Table 2 Statistical source data for Extended Data Fig. 2.

Source Data Extended Data Fig. 3 and Table 3 Statistical source data for Extended Data Fig. 3.

Source Data Extended Data Fig.4 and Table 4 Statistical source data for Extended Data Fig. 4.

Source Data Extended Data Fig. 5 and Table 5 Statistical source data for Extended Data Fig. 5.

## Data Availability

All Visium datasets, including Space Ranger output and high-resolution images, needed to replicate and further build upon the presented analyses have been deposited to BioStudies under accession numbers S-BSST1410 for the human data and S-BSST1409 for the mouse data. These repositories also include sample metadata, processed Seurat/STUtility objects containing deconvolution and clustering results, and cell2location results for all samples. The raw and processed RNA-sequencing data for the mouse samples have been deposited to the GEO under accession number GSE267904. The processed data (Space Ranger output) from the human samples have also been deposited to ArrayExpress under accession number E-MTAB-14121. Due to Swedish law, patient consent and the risk of finding personally identifiable information within the sequencing data, the raw RNA-sequencing read data for the human samples cannot be shared. Source data are provided with this paper.

## Code availability

The code used for downstream data analyses performed in R that was used to produce all analyses and figures presented in the article is freely available at the GitHub repository via https://github.com/lfranzen/spatial-lung-fibrosis, with a mirrored deposition to Zenodo under 10.5281/zenodo.11193764 (ref. ^75^).
