# Supplementary material for: Mapping spatially resolved transcriptomes in human and mouse pulmonary fibrosis
Source: Nat Genet. 2024 Jul 1;56(8):1725–36. doi: 10.1038/s41588-024-01819-2 (PMC11319205; doi:10.1038/s41588-024-01819-2)
Supplement: Supplementary file 1 — Supplementary Notes 1–7, Figs. 1–3, references and legends. [file 41588_2024_1819_MOESM1_ESM.pdf]

nature genetics

Supplementary information https://doi.org/10.1038/s41588-024-01819-2

Mapping spatially resolved transcriptomes in human and mouse pulmonary fibrosis

In the format provided by the authors and unedited

Table of content

Supplementary Notes ***2***
Supplementary Note 1. Extended study motivation and sample size for human data 2
Supplementary Note 2. Extended results of differential expression in IPF alveolar and fibrotic tissue regions 2
Supplementary Note 3. Extended description of human NMF factors 3
Supplementary Note 4. Expected aberrant basaloid cell detection rate in IPF patients 5
Supplementary Note 5. Choice of pulmonary fibrosis animal model 6
Supplementary Note 6. Limitations of the study 6
Supplementary Note 7. Extended methods. 7
Supplementary Figures ***8***
Supplementary References ***11***

Supplementary Notes

Supplementary Note 1. Extended study motivation and sample size for human data

We present an exploratory study aimed at characterizing mechanisms that may underpin idiopathic pulmonary fibrosis (IPF) pathogenesis within the fibrotic niches of the lungs. With highly limited previously generated data of the spatially resolved transcriptome of IPF lungs, we chose to focus our study on a cohort of four IPF patient lungs, with a larger selection of multiple distal lung regions within each patient to capture a variety of histopathological features. These features included minimally remodeled ("normal"-looking) alveolar tissue, dense fibrotic areas, fibroblastic foci, "honeycombing" structures, and immune cell infiltrates. As such, we refrained from any analyses aimed to characterize IPF patient heterogeneity and rather focused on features (histopathological and cell types) that have been previously characterized across larger cohorts of IPF patient lungs.

The number of patient donors included in this study was determined based on tissue availability, RNA quality, histological integrity, as well as constraints of conducting spatially resolved transcriptomic analysis at larger scale. In spatial omics experiments, there are multiple factors to consider when deciding the required number of samples, as outlined in a study by Baker *et al.*[1], that includes the size and number of the method's field of view (FOV), the spatial resolution, and the expected detection rate of any specific cell type or feature. To extract the most information and maintain an unbiased nature of the transcriptomic analyses, the Visium Spatial Gene Expression platform was chosen. With this spatial method, the full polyadenylated transcriptome can be captured, resulting in spatial RNA-sequencing data from thousands of genes within the same tissue section. This allowed for detection of both known and previously unknown transcriptomic signatures in IPF. Further, we chose to primarily explore the interface between less remodeled alveolar tissue and the fibrotic lesions in the distal lung, including fibroblastic foci regions with a focus on the previously identified *KRT5-/KRT17+* aberrant basaloid cell type (expected cell type detection rate in IPF is presented in **Supplementary Note 4**). By increasing the number of FOVs (known as "capture areas" in Visium) per IPF lung, and ensuring their capture of relevant histopathology, we expanded the total area analyzed per patient which allowed us to increase the likelihood for finding signatures of interest.

An equal number of control donors (N=4) was included, where tissue samples from the distal lung parenchyma were analyzed, which allowed for balanced statistical analyses between conditions. Limitations of our study, including sample size and spatial technology, are further stated in **Supplementary Note 6**.

Supplementary Note 2. Extended results of differential expression in IPF alveolar and fibrotic tissue regions

To characterize the minimally injured alveolar compartment of the IPF lung sections and look for early fibrogenic signs, a pseudo-bulk differential expression analysis between annotated "alveolar" and "fibrotic" tissue areas in the IPF samples compared to the HC alveolar regions was performed. A total of 305 and 690 differentially expressed genes (DEGs; adj. p-value < 0.01) were identified in the IPF

alveolar and fibrotic regions, respectively (**Extended Data Fig. 2a,b**, **Supplementary Table 3**). A large overlap of DEGs was observed between these regions, with 223 (95%) upregulated genes and 61 (86%) downregulated genes in IPF alveolar regions also significantly altered in IPF fibrotic regions (**Extended Data Fig. 2c**). The substantial overlap of DEGs points to a common molecular foundation underlying both “early” and advanced stages of IPF within the same lung. This indicates widespread molecular changes present in the pre-fibrotic alveolar tissue within the IPF lungs, even in the absence of apparent tissue remodeling of the lung parenchyma at a histopathological level.

This analysis pinpointed genes of interest in early fibrogenic processes within the alveolar compartment, however, assessing their importance at a spatial level is needed to determine whether they are found adjacent to or distant from to the fibrotic lesions. To delve deeper into the spatial dynamics of the shared upregulated DEGs, we analyzed the correlation between their gene expression and the distance from the fibrotic border into the alveolar regions (**Extended Data Fig. 2d (i)**). The correlation (Pearson) was computed at the 0-500 µm distance and allowed for selection of significantly correlated genes (FDR adj. p-value < 0.05, **Supplementary Table 3**). Ten genes had a decreasing expression moving into the alveolar tissue (negatively correlated), and many of them displayed a peaked expression at the fibrosis side of the border (*CCL18*, *CXCL10*, *CXCL11*, *IDO1*, *MT1X*, *PKP2*, *RAB31*). These genes are largely expressed by cells of myeloid origin and suggests recruitment of immune cells to the edge of fibrotic remodeling. Conversely, 42 DEGs had a positive correlation of gene expression, i.e. increasing expression along the distance from the fibrotic border. Examining the top 10 positively correlated genes, a decrease in gene expression was seen specifically around the border between fibrotic and alveolar tissue. These genes included *AEBP1*, *APOLD1*, *COL1A1*, *COL1A2*, *LTBP1*, and *SPARC*, and their expression patterns may reflect fibroblast-dense regions and ongoing structural alteration within the transitioning alveolar areas. To confirm the indication of various cell types based on gene expression around the fibrotic border, we inspected the inferred cell densities of myofibroblasts, fibroblasts, monocytes, macrophages, and AT1 cells (**Extended Data Fig. 2d bottom panel**). This revealed an accumulation of macrophages and monocytes at the fibrotic side of the border, aligning with the peaked expression of the myeloid-related genes. Additionally, the observed decrease of structural gene expression at the interface coincided with lower densities of myofibroblasts and fibroblasts, further suggesting ongoing remodeling and fibroblast activity within the transitional zone.

Supplementary Note 3. Extended description of human NMF factors

Factor 2 (F2). F2 presents a distinct molecular signature, with its activity predominantly observed in the disease state (**Fig. 2a**). This factor is characterized by a diverse set of genes, including *SLC34A2*, *SFTPB*, and *CTSH*, which are implicated in surfactant metabolism, oxidative stress response, and epithelial cell function. Notably, F2 appears to overlap with areas of honeycombing and fibrosis within IPF lungs, similarly to F5 and F21 (**Supplementary Fig. 1a**).

The enrichment in pathways such as surfactant metabolism and pulmonary interstitial thickening (**Supplementary Table 4**) suggests a role in the aberrant alveolar environment of IPF. The overlap of

F2 with regions of honeycombing and fibrosis further highlights the association of F2 with advanced disease features, pointing towards a signature that captures genes driving fibrotic remodeling and altering pulmonary surfactant balance.

Factor 4 (F4). The top contributing genes to F4, including *PTGDS*, *IGFBP4*, *THY1*, *MMP2*, and a series of collagen genes (e.g., *COL1A1*, *COL3A1*, *COL6A3*) (**Supplementary Table 4**), points towards a molecular network of ECM production, modification, and interaction within fibrotic regions. While both F4 and F14 were highly over-represented in the IPF samples, associated with regions of fibrosis (**Fig. 2a**), and share several contributing genes (**Supplementary Fig. 1b**), the distinct differences between them warrant further discussion. F4 was characterized by an array of ECM-related genes, indicating broad involvement in ECM remodeling and fibrosis, underpinning the structural alterations prevalent in IPF. In contrast, F14 extended beyond the ECM and fibrosis-related gene signature to include keratins such as *KRT7* and *KRT8*, which were not prominently featured in F4. This unique aspect of F14 suggests its potential linkage to the KRT5-/KRT17+ AbBa cell population, a cell type of particular interest due to its potential role in disease progression[2,3].

The spatial correlation patterns of F4 and F14 with cell densities (**Fig. 1e**) offer additional insights into their profiles within the IPF tissue. F4 was associated with both fibroblast and myofibroblast densities, highlighting involvement in a range of fibrotic remodeling processes. On the other hand, F14 correlated with myofibroblast densities, without a significant association with fibroblasts, potentially suggesting a more focused involvement linked to the differentiation state of myofibroblasts and their contribution to ECM deposition and tissue stiffening.

Factor 6 (F6). F6 is enriched in genes encoding components of the immunoglobulin complex (*IGHA1*, *IGKC*, *IGHG* series). The presence of genes such as *CD79A*, a part of the B cell receptor (BCR) complex, along with *JCHAIN* and *MZB1*, which are critical for immunoglobulin assembly and secretion, indicates a presence of B cell involvement embedded within the studied IPF lungs. The correlation of F6 with inferred densities of plasma cells in IPF samples (**Fig. 1e**) moreover points to a contribution of these cells to the immune response within the fibrotic lung environment. This observation is supported by pathway enrichment analyses identifying immunoglobulin complex, complement activation, and B cell receptor signaling pathway among the top significant pathways (**Supplementary Table 4**), implying an interplay between adaptive immunity and fibrosis in IPF.

F6 activity was often found within regions affected by severe tissue remodeling and may therefore indicate a potential role for the humoral immune response in driving or exacerbating fibrotic remodeling. Whether the presence of B/plasma cell infiltrates contributes to or results from fibrosis, warrants further investigations, as does any considerations for therapeutic strategies aimed at modulating B cell activity to influence IPF progression.

Furthermore, the presence of non-immunoglobulin genes such as hypoxia-induced thioredoxin domain-containing protein 5 (*TXNDC5*), prostaglandin D2 synthase (*PTGDS*), and superoxide dismutase 2 (*SOD2*) within F6 suggests a broader involvement in cellular stress responses, potentially linking immune activation with other pathological processes in the disease.

Factor 9 (F9). F9 was identified as a distinct molecular signature within the more mildly remodeled IPF lung tissues, characterized by an expression pattern not found in healthy controls (**Fig. 2a**). This factor contains a specific subset of genes associated with oxidative stress, inflammation, ECM remodeling, and vascular changes, suggesting a joint presence of these biological processes, potentially indicative of early-stage fibrotic alterations or protective responses against fibrosis progression.

The top contributing genes for F9 (**Supplementary Table 4**) include a notable prevalence of metallothioneins (*MT2A*, *MT1M*, *MT1E*, *MT1X*, *MT1A*), which play roles in metal ion homeostasis and protection against oxidative stress[4,5]. Other significant genes such as *IRF1*, *ZFP36*, and *CXCL10* are involved in inflammatory responses[6,7], indicative of ongoing inflammation. Genes like *ICAM1*, *CX3CL1*, and *CXCL10*, along with vascular markers (*CDH5*, *VWF*), point towards changes in vascular integrity and leukocyte trafficking[8-12], further underscoring the involvement of vascular components in the F9 niche.

The presence of genes related to ECM remodeling (*COL4A2*, *COL4A1*, *SERPINH1*, *TIMP3*)[13-15] and fibrotic response modulation (*TGFBI*, *SOD2*, *NAMPT*, *SOCS3*)[16-19] suggests a dynamic ECM turnover and attempts at fibrotic resistance. Additionally, the inclusion of genes like *JUNB*, *IDO1*, and *NFKBIA* highlights the activation of pathways associated with cellular stress responses and immune regulation[20-22].

The spatial co-localization of F9 with endothelial cells and *PLIN2*+ fibroblasts (**Fig. 1e**) underscores a metabolic and vascular remodeling state that may represent an adaptive or early pathogenic response within the IPF lung tissue. Taken together, F9 reveals a complex molecular environment potentially representative of an early fibrogenic stage. The composition of genes associated with oxidative stress, inflammation, ECM remodeling, and vascular changes, distinct from more advanced fibrotic niches, highlights the importance of recognizing and understanding these early molecular alterations.

Supplementary Note 4. Expected aberrant basaloid cell detection rate in IPF patients

The detection rate of the *KRT5-/KRT17+* AbBa cell type in IPF patients is of value to understand in order to estimate the number of samples needed to detect the cell type. Based on previously published scRNA-seq data from IPF lungs[2,3], where the AbBa cell type was described, it is apparent that this cell type appears in a large proportion of the studied IPF lungs albeit at low cell count numbers.

In the Habermann et al. scRNA-seq dataset[3], the *KRT5-/KRT17+* cell type was detected (n cells > 0) in 10 out of 12 (83.3%) IPF patient samples (**Supplementary Fig. 2a,c**). Among these 10 samples, four had less than 10 annotated *KRT5-/KRT17+* cells in total, and all but one had < 2% *KRT5-/KRT17+* cells identified compared to the total number of processed cells.

The Adams et al. scRNA-seq dataset [2] contains a larger IPF cohort of 32 patients. In this study, they annotated an aberrant basaloid cell type that was detected in almost all IPF patient samples (30/32, i.e. 93.8%; **Supplementary Fig. 2b, d**). Nonetheless, the percentage of detected aberrant basaloid cells compared to the total sample cell count was under 2% for all patient samples.

Our present study is in line with the aforementioned scRNA-seq studies in terms of the *KRT5-/KRT17+* AbBa cell type detection rate among IPF donors (marked presence of $hsF14^{hi}$-C0 cluster spots in 3/4 donors, i.e. 75% of donors) (**Supplementary Fig. 2e**). We moreover see a higher abundance of the AbBa $hsF14^{hi}$-C0 cluster spots in IPF donor 3, while lower in IPF donors 1 and 4, likely reflecting a diversity in the number of captured AbBa cells within the studied tissue samples. The number of $hsF14^{hi}$-C0 cluster spots identified per sample was low, not unexpectedly given the rarity of the AbBa cell type. For reference, the highest number of $F14^{hi}$-C0 spots was detected in the IPF donor 3 B1 tissue, where it corresponded to 2.26% of the total number of spots of that tissue section. However, due to differences in input material between studies and the multicellular resolution of a Visium spot, it is important to keep in mind that spot detection rates cannot be used to directly estimate cell type detection rates.

Supplementary Note 5. Choice of pulmonary fibrosis animal model

The single-dose bleomycin mouse model was chosen as the translational model for IPF due to its established reliability in replicating key aspects of human IPF pathology, including the development of fibrosis, inflammation, and aberrant lung remodeling[23]. This model's widespread acceptance in pulmonary research stems from its ability to induce lung injury and fibrotic responses that closely mirror the histopathological and molecular features observed in IPF patients[24]. To inform our choice of time points for analysis in the model, we conducted a pre-study evaluation. This preliminary analysis assessed the expression of alveolar and fibrotic marker genes[25-28] at days 7, 14, 21, and 28 post-bleomycin administration (**Supplementary Fig. 3**). Findings indicated a pronounced alveolar cell depletion and peak pro-fibrotic gene expression by day 7, with a subsequent normalization of alveolar markers and a reduction in fibrosis-associated gene expression towards day 28, suggesting the commencement of resolution beyond day 21. This guided our selection of days 7 and 21 for spatial transcriptomic analysis to capture both the acute response to alveolar injury and the late fibrotic phase before the onset of resolution. By leveraging this model, our study was aimed to enhance our understanding of IPF pathogenesis and characterize its value in translating basic research findings into meaningful clinical insights.

Supplementary Note 6. Limitations of the study

The present study was aimed to provide a view of the global transcriptomic landscape of the fibrotic niches in IPF patient lungs and in lungs from the bleomycin mouse model of pulmonary fibrosis. While the generated dataset is of a large size and exhaustive data analyses have been performed, there are still questions left to be addressed. One such matter is to characterize IPF patient heterogeneity in terms of fibrosis-associated spatial and cellular niches. In the present study, we focused on a smaller number of donor lungs with established IPF and were therefore unable to draw conclusions generalizable to all IPF patients, given the heterogenous nature of the disease. Moreover, the tissue samples examined in this study were collected from patients with late-stage IPF, limiting our ability to detect early-onset signatures of the disease.

We performed analyses to infer the localization of specific cell types within each Visium spatial spot. Nonetheless, a spatial platform that offers cellular or subcellular resolution would be required to fully characterize the location of cell types using established marker molecules.

In our study, we investigated the acute bleomycin mouse model of pulmonary fibrosis, which is widely used to evaluate candidate drugs for IPF. However, it is generally acknowledged that this model does not fully recapitulate the human IPF pathogenesis and disease biology. Further characterization of the relevance of the bleomycin-induced lung fibrosis in mouse to human IPF disease, e.g. by investigating additional timepoints following bleomycin injury and/or repetitive bleomycin administration, may be warranted, but was outside the scope of the present study.

Supplementary Note 7. Extended methods.

Animal monitoring. Animals were monitored and weighed daily throughout the study, with humane endpoints established for signs of distress such as reduced activity, ruffled fur, squinting eyes, huddled posture, piloerection, cyanosis, breathing difficulties, and apathy. Animals experiencing weight loss greater than 15% were to be euthanized immediately to ensure humane treatment. No animals reached these humane endpoints requiring euthanasia in this study. Handling of research animals and BLM challenge were performed as outlined in the main “Methods” section of this paper.

Pre-study evaluation of BLM timepoints. For the time-course qPCR data generation, mouse lung collection was conducted following a similar procedure as for the Visium sample collection. Mice were anesthetized and euthanized, the heart and right lung lobes were tied off, and the right lung lobe was excised. Lung samples were immediately snap-frozen and stored at -80°C until RNA extraction. Total RNA was extracted using RNeasy Plus 96-well kit (Qiagen, 74182). Reverse transcription was performed using the High-Capacity cDNA Reverse Transcription Kit (Applied Biosystems, 4368813) according to manufacturer’s instructions on a Peltier Thermal Cycler PTC-200 (MJ Research). For both the time course study and the current Visium study, qPCR was performed on a QuantStudio7 system (Applied Biosystems) using a custom-designed qPCR array card (TaqMan Low Density Arrays; Applied Biosystems, 4342253). Each sample was assayed with 200 ng of input RNA. Gene expression levels were quantified using the comparative Ct (ΔΔCt) method. Normalization was carried out against an average of five reference genes (*Gapdh, Ppia, B2m, Hprt*, and *Sdha*) and fold change in gene expression (RQ values) was calculated relative to the time-matched vehicle controls.

<u>**Supplementary Figures**</u>

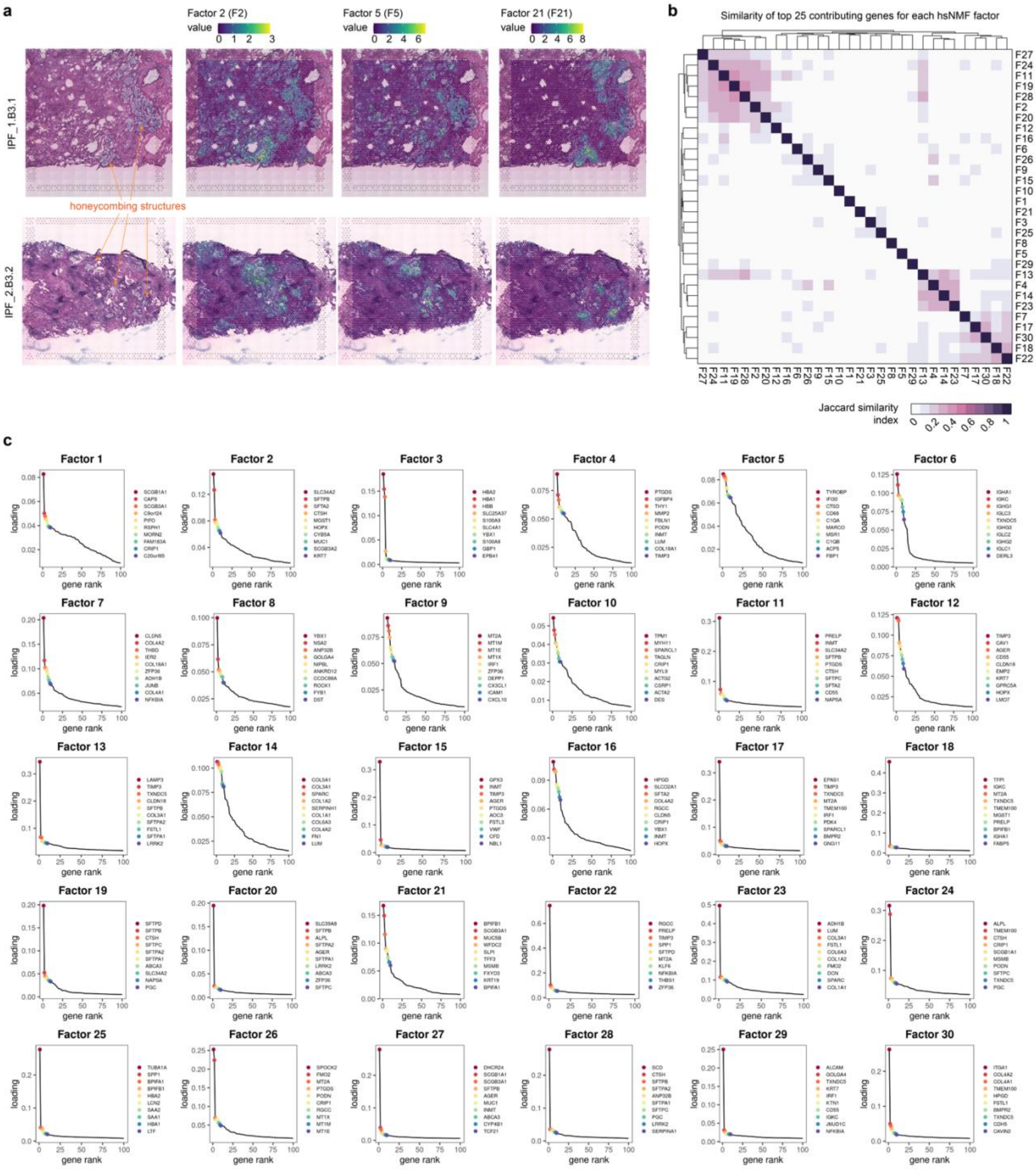

Supplementary Figure 1. Factor activity and gene contributions in IPF lung tissue. a) Spatial visualizations highlighting the activity of factors 2 (F2), 5 (F5), and 21 (F21) in relation to honeycombing regions in IPF lung sections. **b)** Jaccard similarity plot illustrating correlation between all analyzed factors based on their top contributing genes. **c)** Relative weights (loadings) of the top 100 contributing genes (gene rank) for the 30 generated NMF factors, with the top 10 genes highlighted. NMF, non-negative matrix factorization

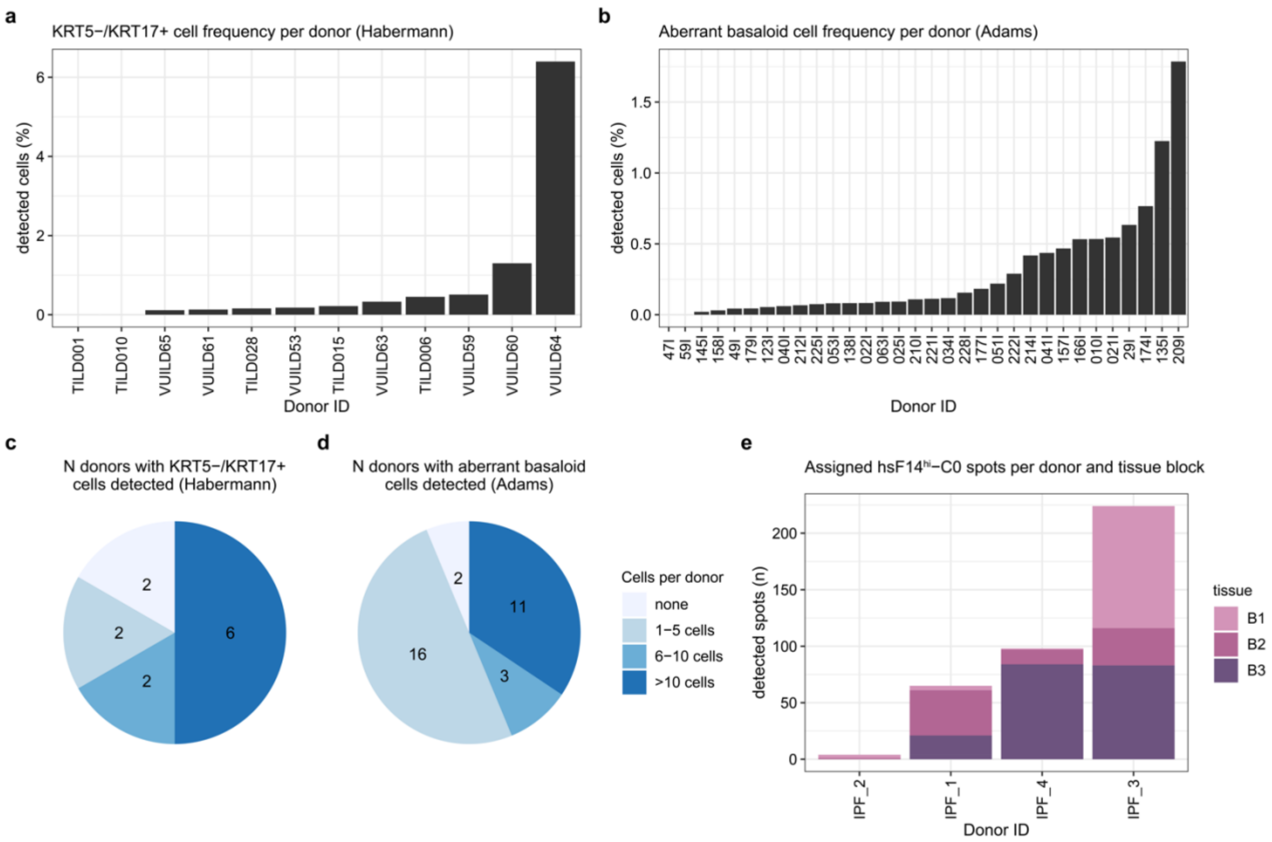

Supplementary Figure 2. Detection of aberrant basaloid cells in IPF lungs. a) Relative frequency of *KRT5-/KRT17+* cells annotated per IPF donor (percentage or total number of cells) in the Habermann et al. (2020) [3] dataset (GSE135893). **b**) Relative frequency of annotated aberrant basaloid cells per IPF donor (percentage of total number of cells) in the Adams et al. (2020) [2] dataset (GSE136831). **c) and d)**, summarized number of IPF donors in which either *KRT5-/KRT17+* cells (*Habermann*, **c**) or aberrant basaloid cells (*Adams*, **d**) were detected, grouped based on the number of cells detected ("none", "1-5 cells", "6-10 cells", or ">10 cells). The number of donors falling into each category is shown for the corresponding slice in the pie charts. **e**) Number of spots in each IPF donor assigned the F14-C0 cluster identity (AbBa-like cluster). The stacked bars are colored based on the tissue block from which the spots originate from. AbBa, aberrant basaloid

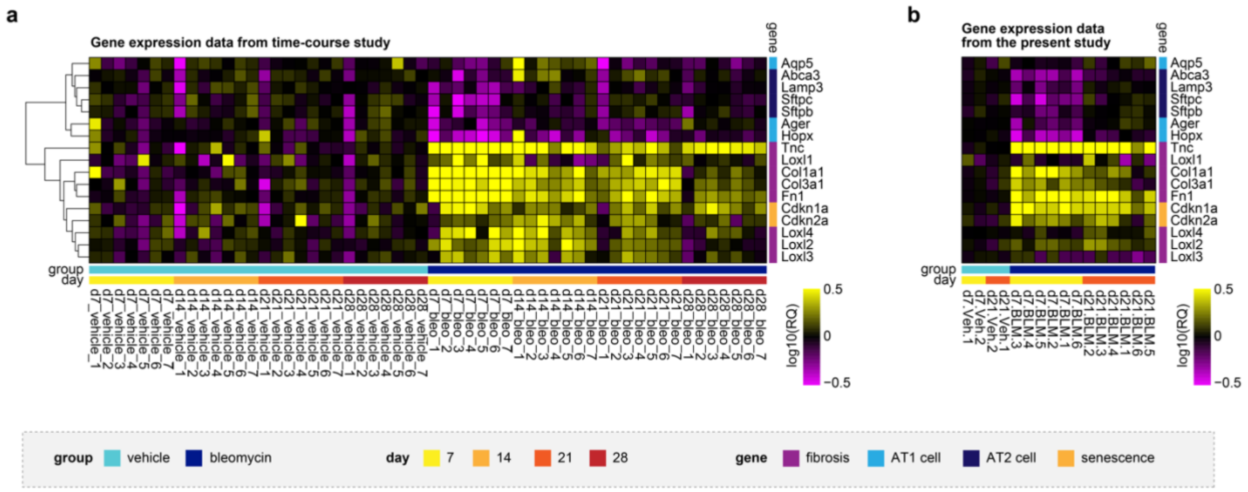

Supplementary Figure 3. Expression of alveolar and fibrotic genes across multiple time points in the single-dose bleomycin mouse model. a) Heatmap visualization of qPCR results displaying relative gene expression in lung tissue samples collected during a time course study of the bleomycin-induced fibrosis mouse model. **b)** Corresponding heatmap of the same qPCR panel applied to the lung tissue samples from the current study on the bleomycin mouse model. Gene expression levels were quantified using the comparative Ct (ΔΔCt) method. Normalization was carried out against an average of five reference genes (*Gapdh, Ppia, B2m, Hprt*, and *Sdha*) and fold change in gene expression (RQ) was calculated relative to the time-matched vehicle controls. RQ: relative quantification ($2^{-\Delta\Delta Ct}$).

Supplementary References

1. Baker, E.A.G., Schapiro, D., Dumitrascu, B., Vickovic, S. & Regev, A. In silico tissue generation and power analysis for spatial omics. *Nat Methods* **20**, 424-431 (2023).
2. Adams, T.S. *et al.* Single-cell RNA-seq reveals ectopic and aberrant lung-resident cell populations in idiopathic pulmonary fibrosis. *Sci Adv* **6**, eaba1983 (2020).
3. Habermann, A.C. *et al.* Single-cell RNA sequencing reveals profibrotic roles of distinct epithelial and mesenchymal lineages in pulmonary fibrosis. *Sci Adv* **6**, eaba1972 (2020).
4. Subramanian Vignesh, K. & Deepe, G.S., Jr. Metallothioneins: Emerging Modulators in Immunity and Infection. *Int J Mol Sci* **18**(2017).
5. Ruttkay-Nedecky, B. *et al.* The role of metallothionein in oxidative stress. *Int J Mol Sci* **14**, 6044-66 (2013).
6. Moore, M.J. *et al.* ZFP36 RNA-binding proteins restrain T cell activation and anti-viral immunity. *Elife* **7**(2018).
7. Newton, R., Shah, S., Altonsy, M.O. & Gerber, A.N. Glucocorticoid and cytokine crosstalk: Feedback, feedforward, and co-regulatory interactions determine repression or resistance. *J Biol Chem* **292**, 7163-7172 (2017).
8. Groom, J.R. & Luster, A.D. CXCR3 ligands: redundant, collaborative and antagonistic functions. *Immunol Cell Biol* **89**, 207-15 (2011).
9. Lenting, P.J., Casari, C., Christophe, O.D. & Denis, C.V. von Willebrand factor: the old, the new and the unknown. *J Thromb Haemost* **10**, 2428-37 (2012).
10. Ley, K., Laudanna, C., Cybulsky, M.I. & Nourshargh, S. Getting to the site of inflammation: the leukocyte adhesion cascade updated. *Nat Rev Immunol* **7**, 678-89 (2007).
11. Umehara, H. *et al.* Fractalkine in vascular biology: from basic research to clinical disease. *Arterioscler Thromb Vasc Biol* **24**, 34-40 (2004).
12. Vestweber, D. VE-cadherin: the major endothelial adhesion molecule controlling cellular junctions and blood vessel formation. *Arterioscler Thromb Vasc Biol* **28**, 223-32 (2008).
13. Brew, K. & Nagase, H. The tissue inhibitors of metalloproteinases (TIMPs): an ancient family with structural and functional diversity. *Biochim Biophys Acta* **1803**, 55-71 (2010).
14. Ito, S. & Nagata, K. Biology of Hsp47 (Serpin H1), a collagen-specific molecular chaperone. *Semin Cell Dev Biol* **62**, 142-151 (2017).
15. Khoshnoodi, J., Pedchenko, V. & Hudson, B.G. Mammalian collagen IV. *Microsc Res Tech* **71**, 357-70 (2008).
16. Epstein Shochet, G., Brook, E., Bardenstein-Wald, B. & Shitrit, D. TGF-β pathway activation by idiopathic pulmonary fibrosis (IPF) fibroblast derived soluble factors is mediated by IL-6 trans-signaling. *Respir Res* **21**, 56 (2020).
17. Gao, F., Kinnula, V.L., Myllärniemi, M. & Oury, T.D. Extracellular superoxide dismutase in pulmonary fibrosis. *Antioxid Redox Signal* **10**, 343-54 (2008).
18. Garcia, A.N. *et al.* eNAMPT Is a Novel Damage-associated Molecular Pattern Protein That Contributes to the Severity of Radiation-induced Lung Fibrosis. *Am J Respir Cell Mol Biol* **66**, 497-509 (2022).

19. Kim, K.K., Sheppard, D. & Chapman, H.A. TGF-β1 Signaling and Tissue Fibrosis. *Cold Spring Harb Perspect Biol* **10**(2018).

20. Hayden, M.S. & Ghosh, S. Shared principles in NF-kappaB signaling. *Cell* **132**, 344-62 (2008).

21. Munn, D.H. & Mellor, A.L. Indoleamine 2,3-dioxygenase and tumor-induced tolerance. *J Clin Invest* **117**, 1147-54 (2007).

22. Shaulian, E. & Karin, M. AP-1 as a regulator of cell life and death. *Nat Cell Biol* **4**, E131-6 (2002).

23. Moore, B.B. & Hogaboam, C.M. Murine models of pulmonary fibrosis. *Am J Physiol Lung Cell Mol Physiol* **294**, L152-60 (2008).

24. Jenkins, R.G. *et al.* An Official American Thoracic Society Workshop Report: Use of Animal Models for the Preclinical Assessment of Potential Therapies for Pulmonary Fibrosis. *Am J Respir Cell Mol Biol* **56**, 667-679 (2017).

25. Beers, M.F. & Moodley, Y. When Is an Alveolar Type 2 Cell an Alveolar Type 2 Cell? A Conundrum for Lung Stem Cell Biology and Regenerative Medicine. *Am J Respir Cell Mol Biol* **57**, 18-27 (2017).

26. Marconett, C.N. *et al.* Cross-Species Transcriptome Profiling Identifies New Alveolar Epithelial Type I Cell-Specific Genes. *Am J Respir Cell Mol Biol* **56**, 310-321 (2017).

27. Ota, C. *et al.* Dynamic expression of HOPX in alveolar epithelial cells reflects injury and repair during the progression of pulmonary fibrosis. *Sci Rep* **8**, 12983 (2018).

28. Peng, R. *et al.* Bleomycin induces molecular changes directly relevant to idiopathic pulmonary fibrosis: a model for "active" disease. *PLoS One* **8**, e59348 (2013).
