# Supplementary material for: Mapping spatially resolved transcriptomes in human and mouse pulmonary fibrosis
Source: Nat Genet. 2024 Jul 1;56(8):1725–36. doi: 10.1038/s41588-024-01819-2 (PMC11319205; doi:10.1038/s41588-024-01819-2)
Supplement: Supplementary file 2 — Reporting Summary [file 41588_2024_1819_MOESM2_ESM.pdf]

nature portfolio

Corresponding author(s): Marianna Stamou
Patrik L Ståhl

Last updated by author(s): May 23, 2024

Reporting Summary

Nature Portfolio wishes to improve the reproducibility of the work that we publish. This form provides structure for consistency and transparency in reporting. For further information on Nature Portfolio policies, see our Editorial Policies and the Editorial Policy Checklist.

Statistics

For all statistical analyses, confirm that the following items are present in the figure legend, table legend, main text, or Methods section.

n/a	Confirmed	
☐	☒	The exact sample size (*n*) for each experimental group/condition, given as a discrete number and unit of measurement
☐	☒	A statement on whether measurements were taken from distinct samples or whether the same sample was measured repeatedly
☐	☒	The statistical test(s) used AND whether they are one- or two-sided<br>*Only common tests should be described solely by name; describe more complex techniques in the Methods section.*
☒	☐	A description of all covariates tested
☐	☒	A description of any assumptions or corrections, such as tests of normality and adjustment for multiple comparisons
☐	☒	A full description of the statistical parameters including central tendency (e.g. means) or other basic estimates (e.g. regression coefficient) AND variation (e.g. standard deviation) or associated estimates of uncertainty (e.g. confidence intervals)
☐	☒	For null hypothesis testing, the test statistic (e.g. *F*, *t*, *r*) with confidence intervals, effect sizes, degrees of freedom and *P* value noted<br>*Give P values as exact values whenever suitable.*
☒	☐	For Bayesian analysis, information on the choice of priors and Markov chain Monte Carlo settings
☒	☐	For hierarchical and complex designs, identification of the appropriate level for tests and full reporting of outcomes
☐	☒	Estimates of effect sizes (e.g. Cohen's *d*, Pearson's *r*), indicating how they were calculated

Our web collection on statistics for biologists contains articles on many of the points above.

Software and code

Policy information about availability of computer code

Data collection: No specific software was used for data collection. The datasets were processed and analyzed using the open source software listed below.

Data analysis: The following software, analysis packages, and code was used:

Space Ranger (v. 1.2.2)
cell2location (v. 0.1)
R (v. 4.0.5 and v. 4.2.3)
STUtility (v. 1.1.1)
Seurat (v. 4.1.1 and v. 4.3.0.1)
NNLM (v. 0.4.4)
DESeq2 (v. 1.30.1)
gprofiler2 (v. 0.2.1)
orthogene (v. 1.4.2)
semla (v. 1.1.6)
ggplot2 (v. 3.4.0)
NicheNet (v. 1.1.1)
multienrichjam (v.0.0.72.900)
Slingshot (v. 1.8)
tradeSeq (v. 1.4.0)
Ingenuity Pathway Analysis (v. 90348151)
Loupe Browser (v. 6)

All the code used for downstream data analyses performed in R (used to produce all figures presented in the article) are available at the GitHub repository https://github.com/lfranzen/spatial-lung-fibrosis and deposited to Zenodo with DOI 10.5281/zenodo.11193764.

For manuscripts utilizing custom algorithms or software that are central to the research but not yet described in published literature, software must be made available to editors and reviewers. We strongly encourage code deposition in a community repository (e.g. GitHub). See the Nature Portfolio guidelines for submitting code & software for further information.

Data

Policy information about availability of data

All manuscripts must include a data availability statement. This statement should provide the following information, where applicable:

- Accession codes, unique identifiers, or web links for publicly available datasets
- A description of any restrictions on data availability
- For clinical datasets or third party data, please ensure that the statement adheres to our policy

Sequencing data was generated using the NovaSeq 6000 (Illumina) system with the following set-up: Read1: 28 bp, Index 1: 10 bp, Index 2: 10 bp, Read2: 90 bp. Human and mouse libraries were sequenced separately over S4 flowcells. FastQ files were processed using the Space Ranger 1.2.2 (10x Genomics) pipeline. Sequencing reads were mapped to their respective reference genomes GRCh38 (human) and mm10 (mouse).

The publicly available single cell data used for cell type deconvolution were retrieved from Gene Expression Omnibus (GEO) database, from studies GSE135893 (human, IPF) and GSE141259 (mouse, bleomycin).

All processed Visium datasets reflecting the "minimum datasets" from the human and mouse lung samples have been deposited to BioStudies under accession numbers S-BSST1410 (https://doi.org/10.6019/S-BSST1410) (human data) and S-BSST1409 (https://doi.org/10.6019/S-BSST1409) (mouse data). The deposited data includes Space Ranger output files, full resolution H&E images, spot alignment files, sample meta data, cell2location results, and Seurat/STUtility objects.

Raw and processed RNA-sequencing data (FASTQ files) for the mouse Visium samples have been deposited to Gene Expression Omnibus (GEO) under accession number GSE267904. The processed Space Ranger output data from all human samples have also been deposited to ArrayExpress under accession number E-MTAB-14121. Raw sequencing data for human samples cannot be made available due to Swedish law, patient consent, and the risk of finding personally-identifiable information within the sequencing data.

Research involving human participants, their data, or biological material

Policy information about studies with human participants or human data. See also policy information about sex, gender (identity/presentation), and sexual orientation and race, ethnicity and racism.

Reporting on sex and gender	Six donors were male and two donors (one control, one IPF) were female. Due to the restricted availability of human biological samples, we were not in a position to select samples based on sex. Information about donor characteristics, including gender, is available in Supplementary Table 1.<br><br>In the analysis of the human Visium data, genes mapped to the X and Y chromosomes were excluded from the downstream analyses in order to avoid biases in the results based on donor sex.
Reporting on race, ethnicity, or other socially relevant groupings	No information regarding race or ethnicity was documented
Population characteristics	Information regarding age, sex, and smoking status of the organ donors have been listed in Supplementary Table 1.
Recruitment	Tissues from lung organ donors with healthy lungs were collected post-mortem and tissue from patient donors with pulmonary fibrosis were collected during lung transplant or resection at the Sahlgrenska University Hospital (Gothenburg Sweden). Lung tissues included in the current study were thereafter selected based on tissue availability and quality.
Ethics oversight	Samples from pulmonary fibrosis patients were acquired with approval by the local human research ethics committee (Gothenburg, permit number 1026-15) and participants gave written informed consent prior to inclusion. Healthy lung tissue was obtained from deceased donors acquired with approval by the local human research ethics committee (Lund, permit number Dnr 2016/317). All donors were anonymized and any derived samples or data cannot be traceable to the donor.

Note that full information on the approval of the study protocol must also be provided in the manuscript.

Field-specific reporting

Please select the one below that is the best fit for your research. If you are not sure, read the appropriate sections before making your selection.

- [x] Life sciences
- [ ] Behavioural & social sciences
- [ ] Ecological, evolutionary & environmental sciences

For a reference copy of the document with all sections, see nature.com/documents/nr-reporting-summary-flat.pdf

Life sciences study design

All studies must disclose on these points even when the disclosure is negative.

Sample size	No statistical method was used to predetermine sample size. Instead, the size was optimized based on practical constraints including availability of suitable tissue, cost considerations, and experimental throughput. This approach is in line with similar spatial transcriptomics studies. For the human samples, a motivation for sample size selection is outlined in Supplementary Note 1.<br><br>For the human study, samples from eight organ donors were used, with an equal split between controls and idiopathic pulmonary fibrosis (IPF) patients, ensuring balanced representation. Within each IPF patient, three distinct tissue samples representing mild, moderate, and severe fibrosis were collected to capture the heterogeneity of the disease across different stages within the same individual. All tissues were analyzed in duplicate sections, with the highest quality samples selected for further analysis. This led to the inclusion of 25 Visium sections representing 16 unique tissues, resulting in 101,075 spatial spots analyzed in the human dataset.<br><br>Mouse lung samples from a total of 18 animals were collected. Tissues were collected at day 7 (bleomycin n=6; control n=3) or day 21 (bleomycin n=6; control n=3) following bleomycin or saline (vehicle control) challenge. The inclusion of 6 mice in the bleomycin groups per timepoint was selected to take hight for the potentially drop-out rate of mice injured by bleomycin, however, all mice fulfilled the inclusion criteria and could be used for the study. Three bleomycin-injured lungs per timepoint were analyzed in technical replicates, resulting in a total of 24 Visium sections. This yielded a total of 91,311 spatial spots across all samples available for analysis in the mouse cohort.<br><br>We aimed for a minimum of three donors/animals per condition and group, consistent with typical experimental design in spatial transcriptomics, to ensure sufficient statistical power without predetermined calculations.
Data exclusions	Visium data was filtered on a spot and gene basis, based on selected thresholds and gene lists outline in detail in the Methods.<br><br>For the human lung tissue Visium data, six replicate sections were excluded from downstream analyses due to poor technical quality (low sequencing mapping results and high degree of lateral diffusion of transcripts). The remaining dataset included data from all eight individuals, and all selected tissues (B1-B3) in the IPF lung samples.
Replication	We implemented several measures to verify the reproducibility of the experimental findings. Both technical and biological replicates were included in the Visium spatial gene expression analysis for human and mouse samples. Specifically, technical replicates consisted of tissue sections processed on different Visium slides. Consistency in results across these technical replicates was confirmed, with comparable data clustering outputs observed, in the cases where both replicates passed the quality control check. Biological replication was achieved using samples from multiple donors or animals, and key findings such as gene expression patterns and spatial distribution were reproducible across these biological replicates where similar histopathological features were captured within the studied tissue section.<br><br>All replication attempts described were successfully reproduced, affirming the reliability of our findings. We have further supported reproducibility by providing open access to all processed data and custom code necessary to replicate our results and figures (see Data and Code Availability statements), ensuring that our findings can be verified by independent analyses.
Randomization	Randomization was not applicable to the human study due to the specific constraints of working with biobanked samples. The samples from IPF patients and healthy controls were selected based on the availability of fresh-frozen lung tissues that met our quality criteria and for which explicit consent had been obtained to conduct genetic studies. As these samples were derived from a biobank (AstraZeneca Biobank), the selection was inherently dependent on what was available and suitable at the time of the study, rather than being randomly assigned from a larger pool.<br><br>For the mouse bleomycin study, conditions allowed for implementation of randomization. Upon arrival, mice were randomly assigned to different treatment groups to ensure that the experimental conditions were evenly distributed and that the results could be generalized across the population studied
Blinding	Blinding was not implemented in the Visium spatial gene expression analyses due to the intrinsic characteristics of the experimental design and the analysis techniques used. The nature of the Visium technology involves spatial mapping of gene expression directly on tissue sections, where the outcomes (i.e., gene expression patterns) are inherently linked to visible, histological features of the tissue. In our experimental setup, the researchers needed to be aware of the specific tissue regions being analyzed to accurately interpret the spatial gene expression data in relation to the tissue morphology. Moreover, the impact of fibrosis in the tissue causes visible histolopathological alterations which makes it impossible for blinding of disease condition in a sample. Blinding would further obscure the link between histopathology and gene expression patterns, potentially compromising the accuracy and relevance of the spatial annotations and the subsequent data interpretation.

Reporting for specific materials, systems and methods

We require information from authors about some types of materials, experimental systems and methods used in many studies. Here, indicate whether each material, system or method listed is relevant to your study. If you are not sure if a list item applies to your research, read the appropriate section before selecting a response.

Materials & experimental systems

n/a	Involved in the study
☒	☐ Antibodies
☒	☐ Eukaryotic cell lines
☒	☐ Palaeontology and archaeology
☐	☒ Animals and other organisms
☒	☐ Clinical data
☒	☐ Dual use research of concern
☒	☐ Plants

Methods

n/a	Involved in the study
☒	☐ ChIP-seq
☒	☐ Flow cytometry
☒	☐ MRI-based neuroimaging

Animals and other research organisms

Policy information about studies involving animals; ARRIVE guidelines recommended for reporting animal research, and Sex and Gender in Research

Laboratory animals	C57BL/6NCrl mice were purchased from Charles River, Germany. At arrival to AstraZeneca R&D Gothenburg (Sweden), the mice were 8 weeks old. After an acclimatization period of 5 days, the study was initiated and the mice were challenged with bleomycin dissolved in saline or saline. Lung samples were thereafter collected at day 7 or day 21 following bleomycin or saline challenge.
Wild animals	The study did not involve wild animals.
Reporting on sex	All mice included in the study (n=18) were of female sex. Sex-based variation in the bleomycin mouse model of pulmonary fibrosis has been seen reported previously, and within AstraZeneca R&D Gothenburg this model has been optimized for female mice.
Field-collected samples	The study did not involve samples collected from the field.
Ethics oversight	Animal handling conformed to standards established by the Council of Europe ETS123 AppA, the Helsinki Convention for the Use and Care of Animals, Swedish legislation, and AstraZeneca global internal standards. All mouse experiments were approved by the Gothenburg Ethics Committee for Experimental Animals in Sweden and conformed to Directive 2010/63/EU. The present study was approved by the local Ethical committee in Gothenburg (EA000680-2017) and the approved site number is 31-5373/11.

Note that full information on the approval of the study protocol must also be provided in the manuscript.

Plants

Seed stocks	*Report on the source of all seed stocks or other plant material used. If applicable, state the seed stock centre and catalogue number. If plant specimens were collected from the field, describe the collection location, date and sampling procedures.*
Novel plant genotypes	*Describe the methods by which all novel plant genotypes were produced. This includes those generated by transgenic approaches, gene editing, chemical/radiation-based mutagenesis and hybridization. For transgenic lines, describe the transformation method, the number of independent lines analyzed and the generation upon which experiments were performed. For gene-edited lines, describe the editor used, the endogenous sequence targeted for editing, the targeting guide RNA sequence (if applicable) and how the editor was applied.*
Authentication	*Describe any authentication procedures for each seed stock used or novel genotype generated. Describe any experiments used to assess the effect of a mutation and, where applicable, how potential secondary effects (e.g. second site T-DNA insertions, mosiacism, off-target gene editing) were examined.*
